# Investigation on the use of ceftazidime-avibactam at a national hospital in Vietnam

**DOI:** 10.1371/journal.pone.0359631

**Published:** 2026-10-13

**Authors:** Khue Dang Minh Van, Han Ngoc Bao Pham, An Thi Thanh Nguyen, Nhi Truc Y. Nguyen, Cam Thi Le Pham, Nhan Thi Thanh Dang, Quynh Thi Huong Bui

**Affiliations:** 1 Department of Clinical Pharmacy, School of Pharmacy, University of Medicine and Pharmacy, Ho Chi Minh City, Vietnam; 2 Department of Pharmacy, Thong Nhat Hospital, Ho Chi Minh City, Vietnam; Dr D Y Patil Vidyapeeth University Dr D Y Patil Medical College Hospital and Research Centre: Dr D Y Patil Medical College Hospital and Research Centre, INDIA

## Abstract

**Objectives:**

To assess the utilization of ceftazidime-avibactam at Thong Nhat Hospital, a national hospital in Vietnam.

**Methods:**

A descriptive cross-sectional study was conducted using retrospective data from medical records of inpatients aged ≥ 18 years who received ceftazidime-avibactam for at least 2 days between January 2023 and March 2025 at Thong Nhat Hospital, Vietnam. The appropriateness of ceftazidime-avibactam use was evaluated based on Thong Nhat Hospital’s antibiotic use guideline. The backward conditional method was used to identify factors associated with patient treatment outcomes.

**Results:**

A total of 101 patients were included, with a median age of 78 years (68−86). Respiratory and bloodstream infections were the most common types, accounting for 84.15% and 44.55% of cases, respectively. Gram-negative bacteria comprised 76.05% of isolates, with *Klebsiella pneumoniae* (26.47%), *Klebsiella aerogenes* (18.50%) and *Pseudomonas aeruginosa* (10.50%) being the most frequent. The susceptibility rate to ceftazidime-avibactam was 71.43% for *Klebsiella pneumoniae*, 95.65% for *Klebsiella aerogenes* and 42.86% for *Pseudomonas aeruginosa*. OXA-48 was the most detected carbapenemase gene (37.84%). The overall appropriateness rate of ceftazidime-avibactam use was 70.30%. The treatment success rate was 39.60%. Treatment in the intensive care unit was associated with decreased treatment success, while comorbid heart failure showed a potential association.

**Conclusions:**

The treatment success rate with ceftazidime-avibactam in this study was relatively low. Continuous surveillance of microbiological profiles and antibiotic resistance, particularly to ceftazidime-avibactam, is essential to better guide the management of drug-resistant Gram-negative infections at the hospital.

## Introduction

Currently, multidrug-resistant Gram-negative infections have become an alarming challenge, significantly limiting treatment options [1]. According to a global report in 2019, the leading bacterial pathogens responsible for deaths associated with antibiotic resistance were *Enterococcus faecium*, *Staphylococcus aureus*, *Klebsiella pneumoniae*, *Acinetobacter baumannii*, *Pseudomonas aeruginosa* and *Enterobacter spp.*, collectively known as the ESKAPE group [2]. Except for *E. faecium* and *S. aureus*, the remaining members of the ESKAPE group are Gram-negative bacteria, highlighting the serious threat posed by Gram-negative infections.

In the past, carbapenems were considered the mainstay for severe Gram-negative infections; however, the emergence of carbapenem-resistant organisms has threatened their effectiveness [1]. In this context, ceftazidime-avibactam is recommended by the Infectious Diseases Society of America (IDSA) as a preferred option for treating carbapenem-resistant Gram-negative infections, including carbapenem-resistant Enterobacterales (defined as resistance to at least one carbapenem or producing carbapenemase) and difficult-to-treat *P. aeruginosa* (DTR – *P. aeruginosa,* defined as resistance to all tested agents, including ceftazidime, cefepime, piperacillin-tazobactam, imipenem-cilastatin, meropenem, ciprofloxacin, levofloxacin and aztreonam) [3,4]. However, in Vietnam, ceftazidime-avibactam has not yet been included in the list of drugs reimbursed by health insurance and remains costly. As a result, ceftazidime-avibactam is primarily used in alternative regimens in infection treatment guidelines in Vietnam, including at Thong Nhat Hospital [5].

Ceftazidime-avibactam was approved for use in Vietnam in 2022. Despite its recent introduction, resistance to ceftazidime-avibactam has already been reported at concerning rates [6,7]. At Bach Mai Hospital, Vietnam (2023), the resistance rates of *K. pneumoniae* and *P. aeruginosa* to ceftazidime-avibactam were at 46.5% and 44%, respectively [6]. At 108 Central Military Hospital, Vietnam (2024), the corresponding rates were at 54.55% and 60%, respectively [7]. IDSA has also warned that bacteria may develop resistance to ceftazidime-avibactam more rapidly than to other newly approved β-lactam/β-lactamase inhibitor combinations [3]. Therefore, rational use of ceftazidime-avibactam is essential to optimize treatment outcomes in patients with drug-resistant Gram-negative infections.

In Vietnam, Gram-negative infections commonly involve the respiratory tract, bloodstream, and urinary tract [8,9]. In a cross-sectional study of ICU-acquired Gram-negative infections, pneumonia was the most frequent infection type, accounting for 70.5% of cases, followed by urinary tract infections and bloodstream infections, at 15.2% and 10.6%, respectively [8]. Another Vietnamese study on ceftazidime-avibactam and ceftolozane-tazobactam use reported that respiratory tract infections, bloodstream infections, and urinary tract infections were the most common indications, accounting for 39.1%, 26.1%, and 25.9% of cases, respectively [9].

Thong Nhat Hospital is a 1200-bed general hospital in southern Vietnam, with a major focus on the care of geriatric patients. Patients treated at Thong Nhat Hospital often have complex clinical conditions and severe infections, in the context of increasing antimicrobial resistance. Therefore, ensuring the rational and safe use of antibiotics, particularly ceftazidime-avibactam, is an important priority in infection management at the hospital. This study was conducted to investigate the current use of ceftazidime-avibactam at Thong Nhat Hospital.

## Methods

### Study design and data collection

A descriptive cross-sectional study was conducted on the medical records of inpatients who received ceftazidime-avibactam between January 2023 and March 2025 at Thong Nhat Hospital, a national hospital in South Vietnam.

Inclusion criteria were inpatients aged ≥ 18 years who were prescribed ceftazidime-avibactam. Exclusion criteria were patients who received ceftazidime-avibactam for less than 2 days and those who absconded or were transferred to another hospital for non-medical reasons. For patients with ≥ 2 hospital admissions involving ceftazidime-avibactam during the study period, only the first admission’s medical records were included.

We collected data from the medical records of patients, including demographic, clinical and laboratory characteristics, microbiological features, ceftazidime-avibactam regimen characteristics and treatment outcomes. The demographic characteristics included age, sex, body mass index (BMI), comorbidities, treatment department, dialysis status, infections and risk factors for multidrug-resistant bacterial infections. BMI was defined as weight (kg) divided by the square of height (m) and was categorized according to the Asia-Pacific cutoff points into four groups: underweight (< 18.5 kg/m^2^), normal weight (18.5–22.9 kg/m^2^), overweight (23.0–25.0 kg/m^2^) and obesity (≥ 25 kg/m^2^) [10]. Clinical and laboratory characteristics included fever, septic shock, altered mental status, coma and renal function parameters (serum creatinine [SCr], creatinine clearance [CrCl], estimated glomerular filtration rate [eGFR]). Microbiological features included isolated pathogens, carbapenem susceptibility, distribution of carbapenemase genotypes and ceftazidime-avibactam susceptibility. Bacterial identification and antimicrobial susceptibility testing were performed using the VITEK 2 automated system. Antimicrobial susceptibility results were interpreted using the Clinical & Laboratory Standards Institute (CLSI) breakpoints integrated into the corresponding instrument software, with the European Committee on Antimicrobial Susceptibility Testing (EUCAST) breakpoints applied only when CLSI breakpoints were unavailable for a specific organism-antimicrobial combination. Carbapenemase genotyping was performed using the Allplex^TM^ Entero-DR Assay (Seegene, Korea). This assay detects carbapenemase genes, including NDM, KPC, OXA-48, VIM, and IMP. Ceftazidime-avibactam regimen characteristics included the type of regimen, duration of therapy, antibiotics used in combination and appropriateness of ceftazidime-avibactam use. Treatment outcome at discharge was extracted from the treating physicians’ documented clinical assessment in the patients’ medical records. According to routine hospital documentation, outcomes were recorded as cured, improved/alleviated, unchanged, worsened, or deceased. For analysis, treatment success was defined as cured or improved/alleviated, whereas treatment failure was defined as unchanged, worsened, or deceased.

### Assessment of the appropriateness of ceftazidime-avibactam use

In this study, the appropriateness of using ceftazidime-avibactam was evaluated based on the Thong Nhat hospital’s guideline [5]. We only assessed the appropriateness of the first ceftazidime-avibactam regimen during treatment period by examining indication and dosage.

Regarding indication, ceftazidime-avibactam use was considered appropriate when all three of the following criteria were met: 1) Ceftazidime-avibactam was used as part of an alternative antibiotic regimen. 2) The patient had at least one infection listed in the ceftazidime-avibactam prescribing information, including complicated intra-abdominal infection, complicated urinary tract infection (including pyelonephritis), hospital-acquired pneumonia (including ventilator-associated pneumonia), or bacteremia that occurs in association with or is suspected to be associated with the above infections. 3) The patient had microbiological characteristics as follow: negative results with no pathogen isolated, or positive results showing carbapenem-resistant Enterobacterales (or detection of a carbapenemase gene such as KPC or OXA-48), or DTR – *P. aeruginosa.* All other cases were considered inappropriate.

Dosage evaluation was only considered when the indication was appropriate. Appropriateness of dosage was defined as dosing and dosing intervals consistent with the ceftazidime-avibactam label, including adjustments based on renal function. For patients with CrCl > 50 mL/min, the appropriate ceftazidime-avibactam dose is 2 g/0.5 g every 8 hours. For those with CrCl 31–50 mL/min, the appropriate dose is 1 g/0.25 g every 8 hours. For patients with CrCl 16–30 mL/min, 6–15 mL/min and for those with end-stage renal disease (including hemodialysis), the appropriate doses are 0.75 g/0.1875 g every 12 hours, 24 hours and 48 hours, respectively [11].

The overall appropriateness of ceftazidime-avibactam use was defined as meeting all criteria, including indication, dosage and dosing interval.

### Statistical analysis

Data were analyzed by IBM SPSS Statistics version 26, R Studio version 2025.05.1 and Microsoft Excel 2016. Continuous variables were described using mean ± standard deviation for normally distributed variables or median (interquartile range) for non-normally distributed variables. Categorical variables were described as frequencies and percentages. Multivariate logistic regression analysis was conducted to identify factors associated with treatment outcomes. The dependent variable was treatment outcome, categorized as success or failure. Independent variables included demographic characteristics (age, comorbidities, Charlson comorbidity index, intensive care unit (ICU) admission, continuous renal replacement therapy, type of infection, septic shock), clinical and laboratory characteristics (altered mental status) and characteristics of ceftazidime-avibactam regimens (monotherapy, duration of therapy, overall appropriateness of use).

The regression analysis was conducted in three steps. First, univariate logistic regression was performed and variables with *p* < 0.2 were selected [12]. Second, multicollinearity was assessed and variables with a variance inflation factor (VIF) < 5 were retained [13]. Finally, eligible variables were included in the multivariate logistic regression model to identify independent associations with treatment outcome. Given the limited sample size, Firth penalized logistic regression was performed as a sensitivity analysis to reduce small-sample bias and assess the robustness of the findings [14]. *P*-values < 0.05 were considered statistically significant.

### Ethics

The protocol of this study was approved by the Institutional Review Board of Thong Nhat Hospital, Ho Chi Minh City, Vietnam (159/2024/CN-BVTN-HDDD, December 23, 2024). The patients’ personal information was kept confidential. All procedures followed the ethical standards set by the institutional and national research committees. The need for informed consent was waived by the Institutional Review Board due to the retrospective nature of the study.

## Results

### Demographic characteristics

A total of 101 patients were included in our study. The majority of the study population were elderly patients (≥ 60 years), with a median age of 78 years (68–86). The median number of comorbidities was 5 (4–6) and the Charlson comorbidity index score was 2 (1–3). Cardiovascular disease was the most common comorbidity (90.10%). Nearly half of the patients required ICU admission (47.53%) and 25.74% required continuous renal replacement therapy (Table 1).

**Table 1:** Patients’ demographic characteristics (n = 101).

Characteristics	Frequency	Percentage
Age (years)	78 (68-86)	
< 60	13	12.87
≥ 60	88	87.13
Sex		
Male	52	51.49
Female	49	48.51
Body mass index (kg/m^2^) (n = 70)	21.7 ± 3.2	
Underweight	10	14.29
Normal weight	38	54.28
Overweight	12	17.14
Obesity	10	14.29
Number of comorbidities	5 (4-6)	
Charlson score	2 (1-3)	
Types of comorbidities		
Cardiovascular Disease	91	90.10
Hypertension	78	77.23
Heart failure	37	36.63
Other cardiovascular diseases^a^	64	63.37
Gastroesophageal reflux disease	40	39.60
Diabetes mellitus	35	34.65
Dyslipidemia	31	30.69
Chronic kidney disease	30	29.70
Cerebrovascular disease	21	20.79
Cancer	5	4.95
Chronic obstructive pulmonary disease	5	4.95
Asthma	2	1.98
Liver failure, cirrhosis	1	0.99
Other comorbidities^b^	55	54.46
Treatment department		
ICU	48	47.52
Non-ICU	53	52.48
Dialysis status		
Continuous renal replacement therapy (CRRT)	26	25.74
Intermittent hemodialysis (IHD)	9	8.91

a Other cardiovascular diseases: atrial fibrillation, ischemic cardiomyopathy, peripheral arterial disease.

b Other comorbidities: epilepsy, gout, Cushing’s syndrome, pulmonary tuberculosis, Parkinson’s disease, benign prostatic hyperplasia, dementia, ureteral stone, renal stone, gallstone, cachexia, chronic adrenal insufficiency, anemia, osteoarthritis, lumbar disc herniation, bladder cancer, pyloric cancer, brain cancer, lung cancer, uterine cancer, gastritis, chronic hepatitis, acute gouty arthritis.

Respiratory tract infections and bloodstream infections were the most frequent types of infections, accounting for 84.15% and 44.55%, respectively. Most patients had risk factors for multidrug-resistant bacterial infections (98.02%). (Table 2)

**Table 2:** Patients’ infection characteristics (n = 101).

Characteristics	Frequency	Percentage
Number of infections	1 (1-2)	
Type of infections		
Septic shock	20	19.80
Sepsis	45	44.55
Respiratory tract infection	85	84.15
Community-acquired pneumonia	26	30.59
Hospital-acquired pneumonia	51	60.00
Ventilator-associated pneumonia	8	9.41
Urinary tract infection	22	21.78
Skin and soft tissue infection	4	3.96
Intra-abdominal infection	3	2.97
Risk factors for multidrug-resistant bacterial infections	99	98.02

### Clinical and laboratory characteristics

The proportions of patients with fever, septic shock, altered mental statusand coma were higher at the time of ceftazidime-avibactam initiation compared to hospital admission. At the time before ceftazidime-avibactam administration, patients’ renal function had markedly deteriorated compared to hospital admission, as indicated by increased SCr and decreased CrCl and eGFR (Table 3)

**Table 3:** Clinical and laboratory characteristics (n = 101).

Characteristics	At admission		Before ceftazidime-avibactam initiation	
	Frequency	Percentage	Frequency	Percentage
Fever	35	34.65	45	44.55
Septic shock	6	5.94	20	19.80
Altered mental status	46	45.54	67	66.33
Coma	11	10.89	23	22.77
Renal function				
SCr (µmol/L)	102 (70.5-185.0)		117.5 (70.8-170.0)	
CrCl (mL/min)	39 (19.0-60.5)		32.5 (20.0-61.5)	
eGFR (mL/min/1,73 m^2^)	64.39 (30.56-85.92)		46.51 (30.35-88.68)	
< 60 mL/min/1,73 m^2^	51 (50.50)		62 (61.39)	
≥ 60 mL/min/1,73 m^2^	50 (49.50)		39 (38.61)	

### Microbiological features

Among 101 patients, 99 (98.02%) had microbiological specimens collected, with 92.93% obtained before the initiation of ceftazidime-avibactam. From these samples, 238 pathogens were isolated, predominantly Gram-negative bacteria (76.05%), with *K. pneumoniae* being the most common (26.47%). (Table 4)

**Table 4:** Isolated pathogens (n = 238).

Organism	Frequency	Percentage
**Gram-negative bacteria**	**181**	**76.05**
*Klebsiella pneumoniae*	63	26.47
*Klebsiella aerogenes*	44	18.50
*Pseudomonas aeruginosa*	25	10.50
*Acinetobacter baumannii*	20	8.40
*Escherichia coli*	12	5.04
*Proteus mirabilis*	4	1.68
*Stenotrophomonas maltophilia*	3	1.26
*Achromobacter xylosoxidans*	3	1.26
*Burkholderia cepacia*	2	0.84
*Burkholderia mallei*	1	0.42
*Klebsiella oxytoca*	1	0.42
*Elizabethkingia meningoseptica*	1	0.42
*Ochrobactrum anthropi*	1	0.42
*Providencia stuartii*	1	0.42
**Gram-positive bacteria**	**21**	**8.82**
*Staphylococcus epidermidis*	4	1.68
*Staphylococcus aureus*	4	1.68
*Staphylococcus haemolyticus*	3	1.26
*Staphylococcus hominis*	3	1.26
*Staphylococcus warneri*	3	1.26
*Staphylococcus capitis*	2	0.84
*Enterococcus gallinarum*	1	0.42
*Streptococcus mitis*	1	0.42
**Fungi**	**30**	**12.61**
*Candida albicans*	13	5.47
*Candida tropicalis*	12	5.04
*Candida auris*	4	1.68
*Candida parasilosis*	1	0.42
**Undetermined pathogen**	**6**	**2.52**

A total of 108 Enterobacterales isolates underwent susceptibility testing, of which 84.26% were carbapenem-resistant. Among 25 *P. aeruginosa* isolates tested, 56.00% were classified as DTR – *P. aeruginosa*. *E. coli* (8 isolates) showed relatively high susceptibility to carbapenems (> 85%). In contrast, *K. pneumoniae* (55 isolates), *K. aerogenes* (40 isolates) and *P. aeruginosa* (25 isolates) exhibited low susceptibility rates (< 25%). (Fig 1)

**Fig 1:**
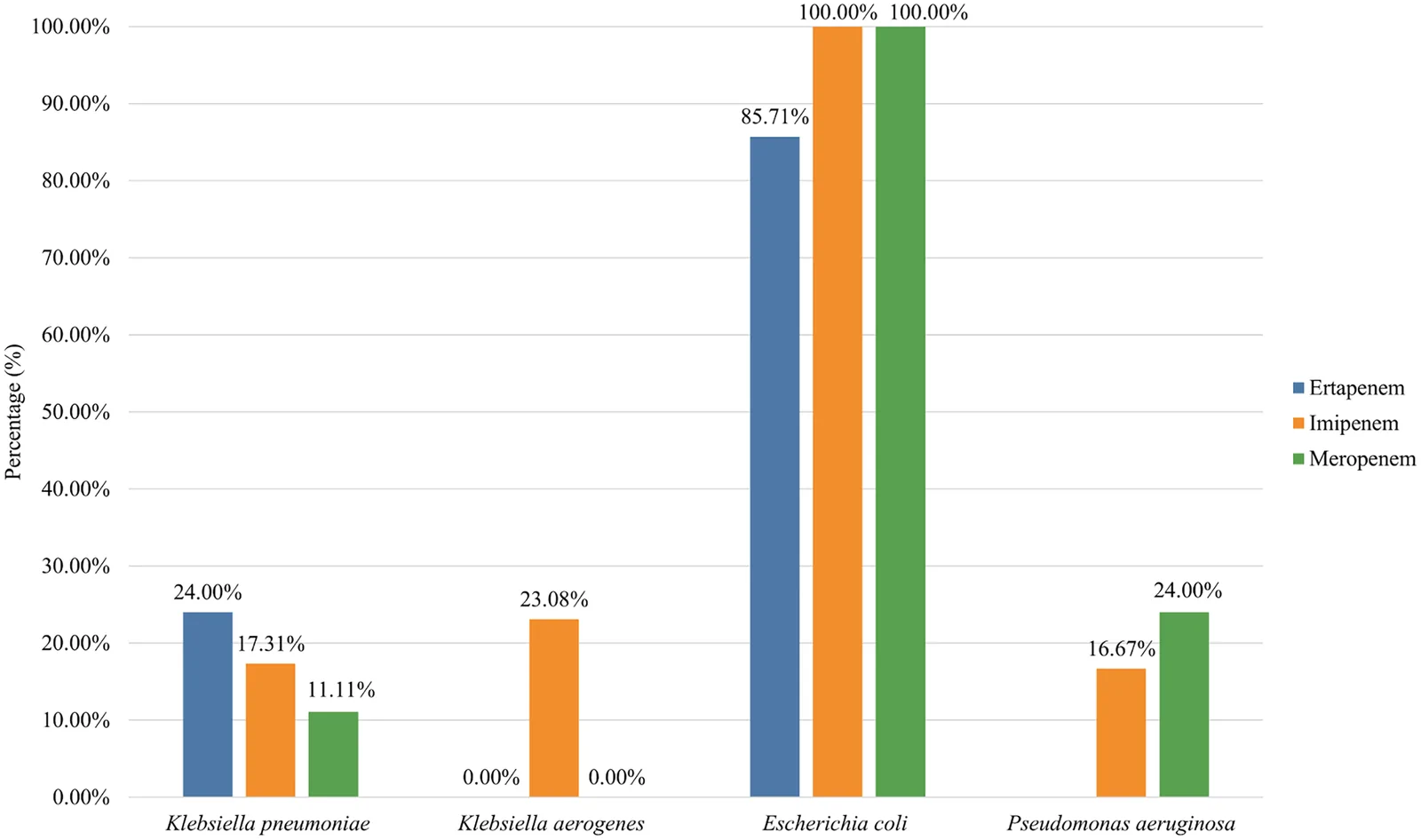
Carbapenem susceptibility of common Gram-negative bacteria.

Among the three most frequently isolated carbapenem-resistant Gram-negative pathogens, *K. aerogenes* (23 isolates) remained highly susceptible to ceftazidime-avibactam (95.65%). *K. pneumoniae* (21 isolates) showed lower susceptibility (71.43%), while *P. aeruginosa* (7 isolates) had the lowest rate (42.86%). (Fig 2)

**Fig 2:**
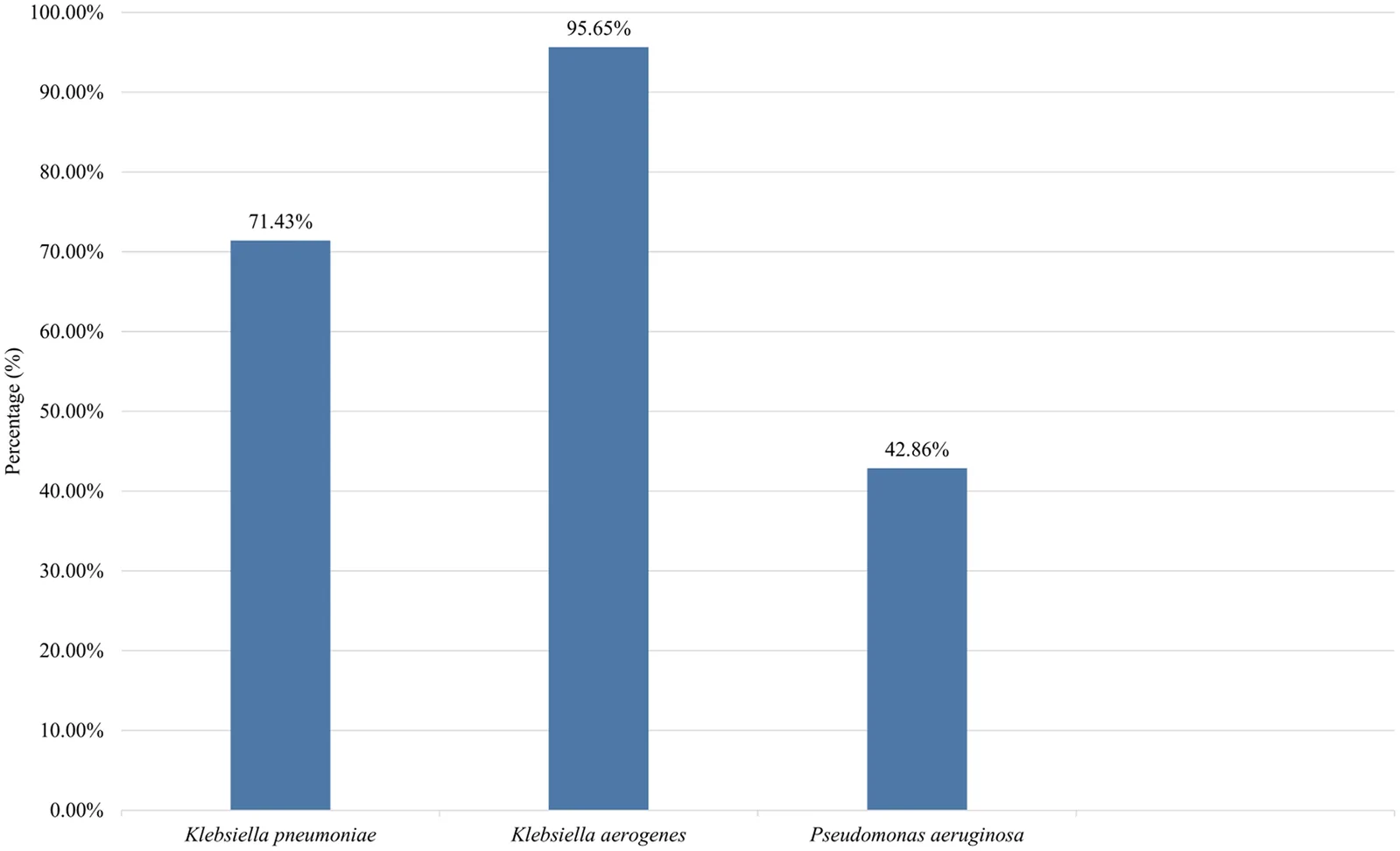
Ceftazidime-avibactam susceptibility of common Gram-negative bacteria.

Regarding the distribution of carbapenemase genotypes, genotyping data were available for only 37 isolates because of the limited availability of carbapenemase gene testing. OXA-48-producing strains accounted for the highest proportion when considering single enzymes (37.84%), while no VIM-producing strains were detected. For combined carbapenemase production, strains co-producing OXA-48 and NDM were the most common (18.92%). (Fig 3)

**Fig 3:**
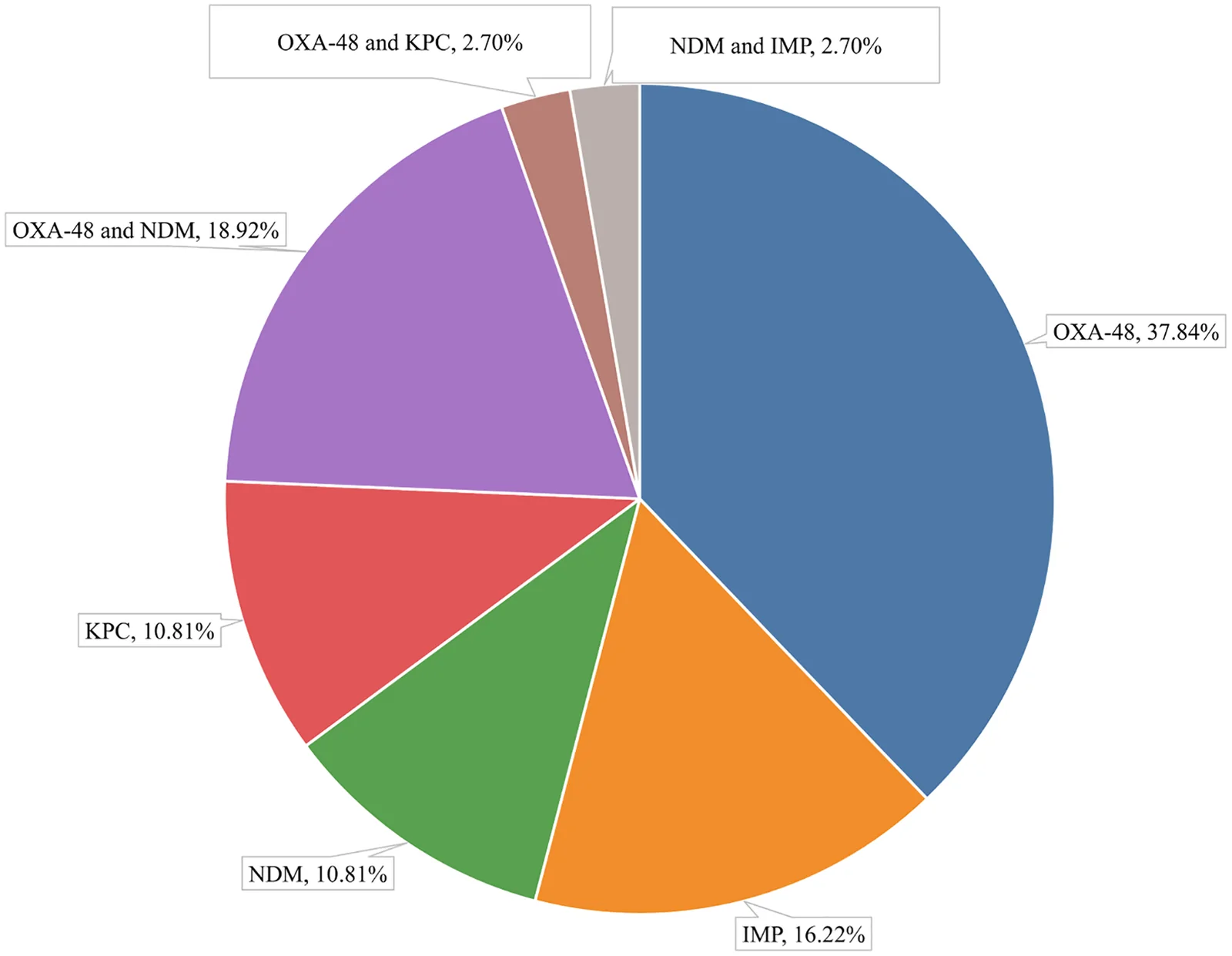
Distribution of carbapenemase genotypes (n = 37).

### Ceftazidime-avibactam regimen characteristics

Most ceftazidime-avibactam regimens were used as an alternative regimen (94.06%). Triple-drug combinations were the most common (49.50%), typically including ceftazidime-avibactam, colistin and an anti-MRSA agent (vancomycin, teicoplanin or linezolid). (Table 5)

**Table 5:** Characteristics of ceftazidime-avibactam regimens (n = 101).

Characteristics	Frequency	Percentage
Type of ceftazidime-avibactam regimen		
Empirical	6	5.94
Replacement	95	94.06
Duration of ceftazidime-avibactam therapy	11 (8-14)	
Antibiotics in combination with ceftazidime-avibactam		
Ceftazidime-avibactam monotherapy	6	5.94
Combination with one additional antibiotic	34	33.66
+ colistin	14	13.86
+ vancomycin/teicoplanin/linezolid	8	7.92
+ amikacin/gentamicin	6	5.94
+ other antibiotics^a^	6	5.94
Combination with two additional antibiotics	50	49.50
+ colistin + vancomycin/teicoplanin/linezolid	20	19.80
+ colistin + fosfomycin	5	4.95
+ fosfomycin + vancomycin/teicoplanin/linezolid	6	5.94
+ fosfomycin + amikacin/gentamicin	5	4.95
+ other antibiotics^b^	14	13.86
Combination with three additional antibiotics	11	10.90
+ colistin + linezolid + fosfomycin	2	1.98
+ colistin + linezolid + aztreonam	1	0.99
+ colistin + linezolid + amikacin	1	0.99
+ other antibiotics^c^	7	6.94

a Other antibiotics: fosfomycin, ticarcillin-clavulanate, tigecycline

b Other antibiotics: colistin + aztreonam/imipenem-cilastatin/meropenem/amikacin/gentamicin/tigecycline, fosfomycin + meropenem/sulfamethoxazole-trimethoprim, amikacin + meropenem/ciprofloxacin/linezolid, vancomycin + sulfamethoxazole-trimethoprim

c Other antibiotics: colistin + ampicillin-sulbactam + aztreonam/vancomycin/sulfamethoxazole-trimethoprim, fosfomycin + ciprofloxacin + linezolid, fosfomycin + vancomycin + sulfamethoxazole-trimethoprim, aztreonam + gentamicin + linezolid, amikacin + levofloxacin + sulfamethoxazole-trimethoprim

The overall appropriate use rate of ceftazidime-avibactam was 70.30%, with 19 cases inappropriate in terms of indication and 11 cases inappropriate in terms of dose or dosing interval. (Tables 6, 7, 8).

**Table 6:** Appropriateness of ceftazidime-avibactam use (n = 101).

Characteristics	Frequency	Percentage
Indication appropriateness	82	81.19
Dose and dosing interval appropriateness (n = 82)	71	86.59
Overall appropriateness	71	70.30

**Table 7:** Reasons for inappropriate use of ceftazidime-avibactam.

Characteristics	Frequency	Percentage
Reasons for inappropriate indication (n = 19)		
Microbiological tests showed pathogens not listed in the evaluation criteria	12	63.16
Empirical treatment with ceftazidime-avibactam before microbiological results were available	6	31.58
Patients infected with pathogens not included in the approved indication	1	5.26
Reasons for inappropriate dose and dosing interval (n = 11)		
Dose higher than appropriate for renal function	11	100

**Table 8:** Inappropriate ceftazidime-avibactam dosing by renal function category at initiation.

Renal function at ceftazidime-avibactam initiation	No. of cases	Administered regimen	Appropriate regimen based on renal function
CrCl 31–50 mL/min	2	Ceftazidime-avibactam 2 g/0.5 g every 8 h	Ceftazidime-avibactam 1 g/0.25 g every 8 h
CrCl 16–30 mL/min	5	Ceftazidime-avibactam 2 g/0.5 g every 8 h	Ceftazidime-avibactam 0.75 g/0.1875 g every 12 h
CrCl 6–15 mL/min	4	Ceftazidime-avibactam 2 g/0.5 g every 8 h	Ceftazidime-avibactam 0.75 g/0.1875 g every 24 h

### Treatment outcome and logistic regression analysis

Overall, the treatment success rate was 39.60%. Six patients died during treatment. (Table 9)

**Table 9:** Treatment outcome (n = 101).

Characteristics	Frequency	Percentage
**Treatment success**	**40**	**39.60**
Cured	1	0.99
Improved/alleviated	39	38.61
**Treatment failure**	**61**	**60.40**
Unchanged	10	9.90
Worsened	45	44.55
Deceased	6	5.94

Compared with patients with treatment success, those with treatment failure more frequently required ICU admission (73.77% vs. 7.50%) and had a higher proportion of septic shock (24.59% vs. 10.00%). Renal function also appeared poorer in the treatment failure group, reflected by higher SCr levels, lower CrCl and eGFR. Regarding microbiological characteristics, ceftazidime-avibactam susceptibility was lower in the treatment failure group than in the treatment success group for *K. pneumoniae* (53.85% vs. 100.00%), *K. aerogenes* (9.10% vs. 100.00%), and *P. aeruginosa* (33.33% vs. 80.00%). (Table 10)

**Table 10:** Clinical, microbiological and treatment characteristics according to treatment outcome.

Characteristics	Treatment success n = 40	Treatment failure n = 61
Age (years)	78 (66.5-85)	78 (69-86)
Body mass index (kg/m^2^)	21.18 ± 3.11	22.13 ± 3.28
Number of comorbidities	5 (4-6)	5 (3-6)
Charlson score	2 (1-3)	2 (1-3)
**Treatment department (%)**		
ICU	7.50	73.77
Non-ICU	92.5	26.23
**Type of infection (%)**		
Respiratory tract infection	77.50	93.44
Sepsis	55.00	37.70
Septic shock	10.00	24.59
Urinary tract infection	35.00	18.03
Skin and soft tissue infection	0.00	9.84
Intra-abdominal infection	5.00	8.20
**Renal function**		
SCr (µmol/L)	98.0 (70.8-122.8)	107.0 (71.0-201.0)
CrCl (mL/min)	43.0 (28.0–59.0)	37.0 (18.0-63.5)
eGFR (mL/min/1,73 m^2^)	66.88 (44.51-81.05)	52.97 (27.84-88.08)
**Ceftazidime-avibactam susceptibility (%)**		
*K. pneumoniae*	100.00	53.85
*K. aerogenes*	100.00	9.10
*P. aeruginosa*	80.00	33.33
**Antimicrobial treatment characteristics (%)**		
Empirical CZA use	10.00	3.28
Replacement CZA us	90.00	96.72
CZA monotherapy	2.50	0.00
Combination with other antibiotics	97.50	100.00
Duration of CZA therapy (days)	13 (11-14)	9 (6-13)
Overall appropriate use	75.00	66.67

One patient had augmented renal clearance at ceftazidime-avibactam initiation, with an estimated CrCl of 208 mL/min. This patient received ceftazidime-avibactam 2 g/0.5 g every 8 h and experienced treatment failure, classified as worsened at discharge.

The results of the univariate logistic regression analysis of candidate variables are presented in Table 11.

**Table 11:** Univariate logistic regression analysis of factors associated with treatment outcome.

Independent variable	OR	95% CI	p-value
Age	0.999	0.970-1.029	0.963
Comorbid heart failure (yes)	0.511	0.216-1.206	0.125
Charlson score	0.911	0.701-1.184	0.486
ICU treatment (yes)	0.029	0.008-0.107	<0.001
Continuous renal replacement therapy (yes)	0.081	0.018-0.368	0.001
Bloodstream infection (yes)	2.019	0.898-4.539	0.098
Respiratory tract infection (yes)	0.327	0.108-0.989	0.048
Urinary tract infection (yes)	2.782	1.056-7.329	0.038
Intra-abdominal infection (yes)	3.158	0.277-36.036	0.355
Septic shock before ceftazidime-avibactam initiation (yes)	0.313	0.096-1.017	0.053
Altered mental status at admission (yes)	1.346	0.604-2.999	0.467
Altered mental status before ceftazidime-avibactam initiation (yes)	0.523	0.226-1.211	0.130
Chronic kidney disease (yes)	0.683	0.280-1.669	0.403
Ceftazidime-avibactam monotherapy (yes)	0.418	0.067-2.621	0.352
Duration of ceftazidime-avibactam therapy	1.138	1.035-1.252	0.008
Overall appropriateness of ceftazidime-avibactam use (yes)	1.808	0.727-4.496	0.202

Variables with p < 0.20 in the univariate logistic regression analysis were selected for multicollinearity assessment, including comorbid heart failure, ICU treatment, continuous renal replacement therapy, bloodstream infection, respiratory tract infection, urinary tract infection, septic shock before ceftazidime-avibactam initiation, altered mental status before ceftazidime-avibactam initiation, and duration of ceftazidime-avibactam therapy.

After multicollinearity assessment, all variables had VIF values < 5 and were therefore retained for inclusion in the multivariable logistic regression model. The following variables were included in the multivariable logistic regression model: comorbid heart failure, ICU admission, continuous renal replacement therapy, bloodstream infection, respiratory tract infection, urinary tract infection, septic shock before ceftazidime-avibactam initiation, altered mental status before ceftazidime-avibactam initiation and duration of ceftazidime-avibactam therapy.

Multivariable logistic regression using the backward method was performed in Table 12. The results showed that comorbid heart failure and ICU treatment were independent predictors of a lower treatment success rate (p < 0.05).

**Table 12:** Factors related to the treatment outcome (n = 101).

Factor	OR	95% CI		p-value
		Upper	Lower	
Comorbid heart failure (Yes)	0.311	0.097	0.995	0.049
Treatment in the ICU (Yes)	0.028	0.007	0.110	< 0.001
Duration of ceftazidime-avibactam therapy	1.114	0.976	1.272	0.110

Abbreviations: OR. odds ratio; CI. confidence interval. Bold values indicate statistically significant results.

In the Firth penalized logistic regression analysis, ICU treatment remained associated with lower odds of treatment success, while comorbid heart failure showed a borderline association but did not reach statistical significance. (Table 13)

**Table 13:** Firth penalized logistic regression sensitivity analysis of factors associated with treatment success (n = 101).

Factor	OR	95% CI		p-value
		Upper	Lower	
Comorbid heart failure (Yes)	0.336	0.104	1.004	0.051
Treatment in the ICU (Yes)	0.036	0.009	0.115	< 0.001
Duration of ceftazidime-avibactam therapy	1.103	0.978	1.265	0.113

## Discussion

Most patients had multiple comorbidities with a median number of 5 comorbid conditions (4–6). Cardiovascular diseases were the most frequent (90.10%), of which 36.63% had heart failure. In addition, 34.65% had diabetes mellitus and 29.70% had chronic kidney disease, both recognized risk factors for multidrug-resistant bacterial infections, particularly in the elderly [15]. Heart failure and chronic kidney disease have been shown to be associated with treatment failure of ceftazidime-avibactam in the study by Trang PND (2025) [9] and the OZAVIE study (2025) [16].

Nearly half of the patients in this study required ICU admission (47.53%), which is comparable to the findings of Sarah Jorgensen (2019) [17] and Calvo-García (2020) [18], with rates of 50.2% and 44.4%, respectively. Both studies also demonstrated an association between ICU treatment and treatment failure [17,18]. These results highlight the complex clinical conditions and severe infections observed in many patients in our study.

Respiratory tract infections were the most common type of infection, observed in 84.15% of patients, followed by bloodstream infections (44.55%) and urinary tract infections (21.78%). All these indications are approved for ceftazidime-avibactam use [11]. Respiratory infections were also reported as the most common type in the study by Tsung Ying Yang (2025) [19] and the OZAVIE study (2025) [16].

Before the initiation of ceftazidime-avibactam, the proportions of patients presenting with fever, septic shock, altered mental status and coma were all higher compared to the time of hospital admission. Notably, 66.33% of patients had altered mental status prior to ceftazidime-avibactam use, which was higher than the rate reported by Minh HQ (2025) (53.39%) [20]. Altered mental status has been demonstrated in previous studies to be associated with treatment failure of ceftazidime-avibactam [20].

In this study, Gram-negative bacteria accounted for the highest proportion of positive isolates among all isolated organisms. Among them, *K. pneumoniae*, *K. aerogenes* and *P. aeruginosa* were the most frequently identified species. Other studies worldwide, such as CAVICOR in Spain (2022) [21], EZTEAM in Europe and Latin America (2023) [22], and Tsung Ying Yang’s study in Taiwan (2025) [19] also reported *K. pneumoniae* as the most prevalent Gram-negative pathogen, consistent with our findings. These results indicate that multidrug-resistant Gram-negative bacteria are spreading globally and have become the major cause of severe infections.

Our results showed that 84.26% of Enterobacterales isolates tested in patients treated with ceftazidim-avibactam were carbapenem-resistant, higher than the 73.6% reported in the study by Sarah Jorgensen (2019) conducted across six healthcare facilities in the United States [17]. Notably, among 40 *K. aerogenes* isolates tested, none remained susceptible to ertapenem or meropenem. In addition, 56.00% of *P. aeruginosa* isolates were classified as DTR. This is consistent with the study by Nam HN at Bach Mai Hospital, Vietnam (2025) [23], which reported a 58.1% rate of DTR – *P. aeruginosa*. In our study, susceptibility rates of *P. aeruginosa* to imipenem and meropenem were 16.67% and 24.00%, respectively, comparable to the 18% reported by Trang PND at the University Medical Center Ho Chi Minh City, Vietnam (2025) [9]. In contrast, the OZAVIE study in France (2025) [16] reported a higher susceptibility rate of *P. aeruginosa* to carbapenems (33.6%), indicating that antimicrobial resistance in Vietnam is more severe than in other regions. The high prevalence of carbapenem resistance in Vietnam may be attributed to inappropriate use of broad-spectrum antibiotics, limited effectiveness of antimicrobial stewardship programs and insufficient infection control measures [24,25].

Marked differences were observed in the susceptibility of *K. pneumoniae*, *K. aerogenes* and *P. aeruginosa* to ceftazidime-avibactam. *K. aerogenes* demonstrated the highest susceptibility (95.65%), followed by *K. pneumoniae* (71.43%), while *P. aeruginosa* showed the lowest rate (42.86%). However, the OZAVIE study in France (2025) reported susceptibility rates of 89.9% for *Klebsiella* spp. and 95.4% for *P. aeruginosa* [16]*.* Similarly, the EZTEAM study conducted in Europe and Latin America (2023) found that 90.9% of multidrug-resistant *Klebsiella* spp. and 96% of multidrug-resistant *Pseudomonas* spp. remained susceptible to ceftazidime-avibactam [22]. These findings suggest that although ceftazidime-avibactam has only been introduced in Vietnam since 2022, resistance appears to have developed more rapidly compared to European and Latin American countries, where the agent has been in use since 2015–2016.

Ceftazidime-avibactam resistance arises through diverse and complex mechanisms. The most common mechanism is the production of metallo-β-lactamases (MBLs) [26]. Resistance may also result from mutations in *bla*KPC, which reduces the ability of avibactam to bind to and inhibit KPC enzyme [26]. The emergence of ceftazidime-avibactam resistance highlights the need for combination treatment strategies in difficult-to-treat resistant infections, as combination therapy may help maximize antimicrobial activity against resistant organisms [27]. In addition, IDSA recommendations support combination approaches, such as ceftazidime-avibactam plus aztreonam, for the first-line treatment of MBL-producing Enterobacterales [3].

Among the 37 bacterial isolates identified with carbapenemase genes, 37.84% produced OXA-48, followed by IMP (16.22%), NDM (10.81%) and KPC (10.81%). These results are consistent with the study by Trang PND at the University Medical Center Ho Chi Minh City, Vietnam (2025), in which class D carbapenemases and MBLs were also predominant [9]. No VIM-producing isolates were identified in our study, although VIM enzymes are reported to be highly prevalent in *P. aeruginosa* worldwide [28,29]. This may be due to the relatively small sample size of our study. Geographic differences between continents may also contribute to variations in carbapenemase genotypes [30], as both the studies by Trang PND at the University Medical Center Ho Chi Minh City (2025) [9] and Anh NT at Hanoi Medical University Hospital (2024) [31] also reported no VIM-producing strains.

When considering combined carbapenemase production, the most common finding was the coexistence of OXA-48 and NDM (18.92%). This result is similar to that of the study by Trang PND (2025) [9], where OXA-48 and NDM co-production accounted for the highest proportion (21.7%). The similarity in carbapenemase distribution patterns between the two studies may be explained by geographical factors, as both Thong Nhat Hospital and the University Medical Center Ho Chi Minh City are located in southern Vietnam. In addition, several studies conducted in Asian countries have shown that the most common carbapenemases detected in *K. pneumoniae* are MBLs (mainly NDM) and OXA-48 [32–34]. However, these results differ from studies conducted in the Americas and Europe, where KPC and OXA-48 were the predominant carbapenemases [32].

Before the initiation of ceftazidime-avibactam, 94.06% of patients in our study were prescribed other antibiotic regimens. However, studies conducted worldwide have reported lower proportions of patients receiving ceftazidime-avibactam as a replacement therapy. Specifically, the OZAVIE study (2025) [16], the study by Subhash Todi (2024) [35] and the EZTEAM study (2023) [22] reported proportions of 71.5%, 66.7% and 76.9%, respectively. The difference may be explained by the fact that ceftazidime-avibactam is currently considered a replacement antibiotic for multidrug-resistant infections in Vietnam [5], whereas international treatment guidelines recommend ceftazidime-avibactam as a preferred option [3].

In our study, ceftazidime-avibactam was most frequently used in combination with two other antibiotics (49.50%) and only 5.94% of patients received ceftazidime-avibactam monotherapy. In contrast, the OZAVIE study (2025) reported that 77.8% of patients received ceftazidime-avibactam as monotherapy [16]. This difference in regimen patterns may be attributed to variations in antibiotic stewardship experience between Vietnam and European countries, as well as differences in local resistance epidemiology. In Vietnam, where multidrug-resistant pathogens are highly prevalent and resistance develops rapidly, combination therapy is consistent with formal guidelines and is considered a strategy to enhance treatment effectiveness [5].

The most common triple-therapy regimen in our study consisted of ceftazidime-avibactam combined with colistin or fosfomycin and a third agent active against methicillin-resistant *S. aureus* (vancomycin, teicoplanin or linezolid). For dual therapy, the most common regimen was ceftazidime-avibactam combined with colistin. In contrast, the studies by Subhash Todi (2024) [35] and EZTEAM (2023) [22] reported different findings, as colistin was the second most commonly combined agent, after aztreonam. Globally, aztreonam is widely used due to its activity against MBL-producing Enterobacterales and has been included in international guidelines [3]. However, in Vietnam, the combination of ceftazidime-avibactam and aztreonam has not yet been widely adopted because aztreonam is not officially available in the formularies of many hospitals, including Thong Nhat Hospital. This explains why aztreonam plays a key role as a partner drug in international studies but has not assumed the same role in Vietnam.

A total of 81.19% of patients were appropriately prescribed ceftazidime-avibactam. The main reasons for inappropriate use were microbiological results showing pathogens outside the indication (most commonly *A. baumannii*) or empirical use of ceftazidime-avibactam before microbiological results were available. According to the IDSA guideline, ceftazidime-avibactam is not recommended for the treatment of *A. baumannii* infections [3]. In addition, patients with inappropriate doses in our study had not received ceftazidime-avibactam adjusted for renal function at the time of administration.

In our study, the overall appropriateness rate of ceftazidime-avibactam use was 70.30%, which was lower than that reported in the study by Trang PND (2025) [9], at 81.7%. This difference may be attributed to variations in the criteria used to assess indication appropriateness. Our study evaluated appropriateness based on Thong Nhat Hospital’s guideline, in which ceftazidime-avibactam was considered appropriate only when all the following criteria were met: use as an alternative regimen, treatment of approved infections and either negative microbiological results or isolation of carbapenem-resistant Enterobacterales or DTR – *P. aeruginosa*. In contrast, in the study by Trang PND [9], ceftazidime-avibactam was considered appropriate only based on whether patients had approved infections, with no additional criteria required.

In this study, ceftazidime-avibactam therapeutic drug monitoring (TDM) was not performed; therefore, plasma drug concentrations and PK/PD target attainment could not be assessed. However, Fresán (2023) reported that TDM of ceftazidime-avibactam may support dose reduction and potentially reduce antimicrobial-related adverse effects and treatment costs [36]. In addition, Gatti (2023) showed that optimized joint PK/PD target attainment of continuous-infusion ceftazidime-avibactam may support microbiological eradication in difficult-to-treat Gram-negative infections [37]. These findings highlight the potential value of TDM-guided dose optimization, especially in patients with renal impairment, renal replacement therapy, or augmented renal clearance.

The treatment success rate in our study population was 39.60%, which is relatively low. This finding may be explained by the fact that most patients were elderly, had multiple comorbidities and suffered from severe infections caused by multidrug-resistant bacteria, all of which are factors that increase the risk of treatment failure [15,38].

In our study, only one patient had augmented renal clearance and experienced treatment failure despite receiving the standard ceftazidime-avibactam regimen. Although no causal conclusion can be drawn from a single case, this finding highlights the importance of considering high renal clearance when optimizing ceftazidime-avibactam therapy.

ICU treatment was significantly associated with lower odds of treatment success in the multivariable logistic regression analysis (OR = 0.028, 95% CI: 0.007–0.110, p < 0.001). This association remained statistically significant in the Firth penalized logistic regression sensitivity analysis (OR = 0.036, 95% CI: 0.009–0.115, p < 0.001), suggesting that ICU treatment was a robust factor associated with lower treatment success in our study. This finding is consistent with the findings of Sarah Jorgensen (2019) [17] and Calvo-García (2020) [18]. Patients requiring ICU admission typically present with more severe clinical conditions, multiorgan dysfunction and a higher risk of multidrug-resistant infections, resulting in poorer treatment outcomes compared to others. Furthermore, factors such as the complex antibiotic resistance profile and higher rates of invasive procedures among ICU patients may also contribute to the increased risk of treatment failure [39,40].

In the multivariable logistic regression analysis, comorbid heart failure was significantly associated with a reduced likelihood of treatment success (OR = 0.311, 95% CI: 0.097–0.995, p = 0.049). However, in the Firth penalized logistic regression sensitivity analysis, this association no longer reached statistical significance, although it remained borderline (OR = 0.336, 95% CI: 0.104–1.004, p = 0.051). Therefore, comorbid heart failure should be interpreted as a potential factor associated with lower treatment success rather than a definitive independent factor in our study. Previous studies have also reported an association between heart failure and poorer treatment outcomes [9,16]. This may be because patients with heart failure generally have weaker resistance and are more likely to have additional comorbidities, which may increase the risk of developing severe infections and poor treatment response [41].

Our findings have important implications for antimicrobial stewardship, particularly at Thong Nhat Hospital. Timely microbiological diagnosis should be prioritized before ceftazidime-avibactam initiation or as early as possible after initiation, as microbiological results are essential for the judicious use of this new antimicrobial [42]. In addition, renal function should be carefully assessed at treatment initiation and throughout therapy to ensure appropriate dose adjustment, particularly in patients with renal impairment. Finally, ceftazidime-avibactam use should be guided by local microbiological data, susceptibility results and approved indications to preserve its activity and minimize the emergence of further resistance [3,43].

### Limitation

This study had several limitations.

First, the data were retrospectively collected from archived medical records, which may have resulted in incomplete documentation of key variables, particularly laboratory response indicators and therefore limited the ability to comprehensively assess factors related to antibiotic use.

Second, the research was conducted at a single center with a relatively small sample size, which may not fully capture the diversity of the patient population. This also limits the precision of conclusions regarding antimicrobial resistance patterns and the clinical effectiveness of ceftazidime-avibactam in the hospital setting. Hence, further studies are warranted to address these limitations and provide comprehensive insights into this subject.

Moreover, in our study, carbapenemase genotyping was available for only 37 isolates. Although the hospital protocol recommended genotyping for isolates resistant to at least one carbapenem, the test was not reimbursed by health insurance, limiting its routine implementation. As a result, the genotyped isolates may not fully represent all carbapenem-resistant isolates in the study population, and the distribution of carbapenemase genes should be interpreted as descriptive findings from the tested subset.

## Conclusion

It is necessary to strengthen monitoring and evaluation of the appropriate use of ceftazidime-avibactam to optimize treatment, particularly in patients treated in the ICU. Comorbid heart failure should be interpreted cautiously as a potential factor associated with lower treatment success. Ongoing surveillance of microbiological patterns and resistance, particularly to ceftazidime-avibactam, is needed to guide the management of drug-resistant Gram-negative infections.

## Supporting information

S1 File Dataset. (XLSX)

## References

[1] Doi Y, Bonomo RA, Hooper DC, Kaye KS, Johnson JR, Clancy CJ, et al. Gram-negative bacterial infections: Research priorities, accomplishments, and future directions of the antibacterial resistance leadership group. Clin Infect Dis. 2017;64(suppl_1):S30–5. doi: 10.1093/cid/ciw829 PMC5848311 28350901

[2] Antimicrobial Resistance Collaborators. Global burden of bacterial antimicrobial resistance in 2019: A systematic analysis. Lancet. 2022;399(10325):629–55. doi: 10.1016/S0140-6736(21)02724-0 35065702 PMC8841637

[3] Tamma PD, Aitken SL, Bonomo RA, Mathers AJ, van Duin D, Clancy CJ. Infectious diseases society of America 2022 guidance on the treatment of extended-spectrum β-lactamase Producing Enterobacterales (ESBL-E), Carbapenem-Resistant Enterobacterales (CRE), and *Pseudomonas aeruginosa* with Difficult-to-Treat Resistance (DTR-*P. aeruginosa*). Clin Infect Dis. 2022;75(2):187–212. doi: 10.1093/cid/ciac268 35439291 PMC9890506

[4] National Healthcare Safety Network C for DC and Prevention. Antibiotic resistance patient safety atlas: phenotypic definitions. 2016. https://www.cdc.gov/hai/pdfs/patient-safety-atlas/AR-Patient-Safety-AtlasPhenotype-Definitions.pdf

[5] Quynh BTH, Thanh LD. Guidelines for antibiotic use. Medical Publishing House; 2022.

[6] Nhung PH, Huong MTL. Antibiotic susceptibility of common bacterial species causing bloodstream infections isolated at Bach Mai Hospital in 2023. J Med Res. 2024;178(5):70–81. doi: 10.52852/tcncyh.v178i5.2436

[7] Thau NS, Tuan NV, The NT, et al. Antibiotic resistance of hospital-acquired infection pathogens in the department of infectious diseases intensive care at 108 military central hospital from January 2024 to September 2024. J Clin Med Pharm. 2024;19(7). doi: 10.52389/ydls.v19i7.2448

[8] Phong DQ, Vinh VH, Van DH, Hieu TM, Huong TTM, Thuong LT, et al. Burden and resistance of Gram-negative pathogens in ICU-acquired infections in Vietnam: A cross-sectional study. Iran J Microbiol. 2026;18(1):32–40. doi: 10.18502/ijm.v18i1.20904 42112297 PMC13156818

[9] Phan NDT, Trần NPM, Đặng NDT. Investigation on the use of ceftazidime-avibactam and ceftolozane-tazobactam at the University of Medicine and Pharmacy, Ho Chi Minh City. Y HOC TP HO CHI MINH. 2025;1. doi: 10.32895/hcjm.p.2025.09.01

[10] Weir CB, Jan A. BMI classification percentile and cut off points. StatPearls. Treasure Island (FL): StatPearls Publishing; 2024.

[11] Zavicefta 2 g/0.5 g powder for concentrate for solution for infusion. 2022.

[12] Malhotra RK. Errors in the use of multivariable logistic regression analysis: An empirical analysis. Indian J Community Med. 2020;45(4):560–2. doi: 10.4103/ijcm.IJCM_16_20 33623224 PMC7877407

[13] Kim JH. Multicollinearity and misleading statistical results. Korean J Anesthesiol. 2019;72(6):558–69. doi: 10.4097/kja.19087 31304696 PMC6900425

[14] Suhas S, Manjunatha N, Kumar CN, Benegal V, Rao GN, Varghese M, et al. Firth’s penalized logistic regression: A superior approach for analysis of data from India’s National Mental Health Survey, 2016. Indian J Psychiatry. 2023;65(12):1208–13. doi: 10.4103/indianjpsychiatry.indianjpsychiatry_827_23 38298875 PMC10826871

[15] Alsehemi AF, Alharbi EA, Alammash BB, Alrais AI, Elbadawy HM, Alahmadi YM. Assessment of risk factors associated with multidrug-resistant organism infections among patients admitted in a tertiary hospital - A retrospective study. Saudi Pharm J. 2023;31(6):1084–93. doi: 10.1016/j.jsps.2023.03.019 37293381 PMC10244471

[16] Piroth L, Vitrat V, Moing VL, Bret P, Brault Y, Greenwood W, et al. Real-world use, effectiveness, and safety of ceftazidime-avibactam: Results of the French cohort OZAVIE. Infect Dis Now. 2025;55(2):105036. doi: 10.1016/j.idnow.2025.105036 39929301

[17] Jorgensen SCJ, Trinh TD, Zasowski EJ, Lagnf AM, Bhatia S, Melvin SM. Real-world experience with Ceftazidime-Avibactam for multidrug-resistant gram-negative bacterial infections. Open Forum Infect Dis. 2019;6(12):ofz522. doi: 10.1093/ofid/ofz522 PMC6934163 31890725

[18] Calvo-Garcia A, Urriaga IBZ, Ramírezherráiz E, Pérezabánades M, Sáezbéjar C, Aladrón MOB, et al. Ceftazidime-avibactam: effectiveness and safety in the clinical practice. A third hospital level experience. 2020.

[19] Yang T-Y, Huang C-T, Liu P-Y, Lin Y-T, Huang Y-S, Chang P-H, et al. Real-world use and treatment outcomes of ceftazidime-avibactam in gram-negative bacterial infection in Taiwan: A multicenter retrospective study. J Infect Public Health. 2025;18(6):102735. doi: 10.1016/j.jiph.2025.102735 40112565

[20] Minh HQ, Nga VTT, Tu HTC, et al. Efficacy of ceftazidime-avibactam in the treatment of bacterial secondary infection in COVID-19 patients at Hospital for Tropical Diseases. Vietnam J Infect Diseases. 2025;1(49):19–26. doi: 10.59873/vjid.v1i49.434

[21] Castón JJ, Cano A, Pérez-Camacho I, Aguado JM, Carratalá J, Ramasco F, et al. Impact of ceftazidime/avibactam versus best available therapy on mortality from infections caused by carbapenemase-producing Enterobacterales (CAVICOR study). J Antimicrob Chemother. 2022;77(5):1452–60. doi: 10.1093/jac/dkac049 35187577

[22] Soriano A, Montravers P, Bassetti M, Klyasova G, Daikos G, Irani P, et al. The use and effectiveness of ceftazidime-avibactam in real-world clinical practice: EZTEAM study. Infect Dis Ther. 2023;12(3):891–917. doi: 10.1007/s40121-023-00762-9 PMC9912999 36763243

[23] Nam NH, Tuan DQ, Cuong BV. Antimicrobial susceptibility of Pseudomonas aeruginosa strains causing pneumonia in the intensive care unit of Bach Mai Hospital, 2024. Vietnam Med J. 2025;547(2). doi: 10.51298/vmj.v547i2.12959

[24] Vu TVD, Choisy M, Do TTN, Nguyen VMH, Campbell JI, VINARES consortium. Antimicrobial susceptibility testing results from 13 hospitals in Viet Nam: VINARES 2016–2017. Antimicrob Resist Infect Control. 2021;10(1):78. doi: 10.1186/s13756-021-00937-4 PMC8112055 33971969

[25] GARP-Vietnam National Working Group. Situation analysis: antibiotic use and resistance in Vietnam. Washington D.C.: The Center for Disease Dynamics, Economics & Policy; 2010. https://cddep.org/wpcontent/uploads/2017/08/garp-vietnam_sa.pdf

[26] Li J, Wang H, Liu Z, Xia Y, Hu Y, Wang H, et al. Exposure to ceftazidime-avibactam induced antimicrobial resistance and enhanced virulence in a *Klebsiella pneumoniae* strain. iScience. 2025;28(11):113844. doi: 10.1016/j.isci.2025.113844 41280666 PMC12636361

[27] Mikhail S, Singh NB, Kebriaei R, Rice SA, Stamper KC, Castanheira M, et al. Evaluation of the synergy of ceftazidime-avibactam in combination with meropenem, amikacin, aztreonam, colistin, or fosfomycin against well-characterized multidrug-resistant *Klebsiella pneumoniae* and *Pseudomonas aeruginosa*. Antimicrob Agents Chemother. 2019;63(8):e00779-19. doi: 10.1128/AAC.00779-19 31182535 PMC6658738

[28] Horcajada JP, Montero M, Oliver A, Sorlí L, Luque S, Gómez-Zorrilla S, et al. Epidemiology and treatment of multidrug-resistant and extensively drug-resistant *Pseudomonas aeruginosa* infections. Clin Microbiol Rev. 2019;32(4):e00031-19. doi: 10.1128/CMR.00031-19 31462403 PMC6730496

[29] Reyes J, Komarow L, Chen L, Ge L, Hanson BM, Cober E, et al. Global epidemiology and clinical outcomes of carbapenem-resistant *Pseudomonas aeruginosa* and associated carbapenemases (POP): A prospective cohort study. Lancet Microbe. 2023;4(3):e159–70. doi: 10.1016/S2666-5247(22)00329-9 36774938 PMC10016089

[30] Peirano G, Matsumura Y, Adams MD, Bradford P, Motyl M, Chen L. Genomic epidemiology of global carbapenemase-producing *Enterobacter* spp., 2008–2014. Emerg Infect Dis. 2018;24(6):1010–9. doi: 10.3201/eid2406.171648 PMC6004858 29774858

[31] Anh NT, Hieu VN, Tuyet TT, Nhung PH. Antibiotic susceptibility and carbapenemase gene profiles of *Pseudomonas aeruginosa* isolates at Hanoi Medical University Hospital, 2019-2022. J Med Res. 2024;178(5):61–9. doi: 10.52852/tcncyh.v178i5.2408

[32] Nordmann P, Poirel L. Epidemiology and diagnostics of carbapenem resistance in gram-negative bacteria. Clin Infect Diseases. 2019;69(Supplement_7):S521–8. doi: 10.1093/cid/ciz824 PMC6853758 31724045

[33] Paveenkittiporn W, Lyman M, Biedron C, Chea N, Bunthi C, Kolwaite A. Molecular epidemiology of carbapenem-resistant *Enterobacterales* in Thailand, 2016–2018. Antimicrob Resist Infect Control. 2021;10(1):88. doi: 10.1186/s13756-021-00950-7 PMC8180034 34090537

[34] Garg A, Garg J, Kumar S, Bhattacharya A, Agarwal S, Upadhyay GC. Molecular epidemiology and therapeutic options of carbapenem-resistant Gram-negative bacteria. Indian J Med Res. 2019;149(2):285–9. doi: 10.4103/ijmr.IJMR_36_18 31219096 PMC6563745

[35] Todi S, Sathe P, V R, Swaminathan S, Talwar D, Prayag P. Real-world evidence on use of ceftazidime-avibactam in the management of gram-negative infections: A retrospective analysis. Cureus. 2024. doi: 10.7759/cureus.70234 PMC11512339 39463643

[36] Fresan D, Luque S, Benítez-Cano A, Sorlí L, Milagro Montero M, De-Antonio M, et al. Pharmacokinetics/pharmacodynamics and therapeutic drug monitoring of ceftazidime/avibactam administered by continuous infusion in patients with MDR Gram-negative bacterial infections. J Antimicrob Chemother. 2023;78(3):678–83. doi: 10.1093/jac/dkac439 36626402

[37] Gatti M, Rinaldi M, Bonazzetti C, Gaibani P, Giannella M, Viale P, et al. Could an optimized joint pharmacokinetic/pharmacodynamic target attainment of continuous infusion ceftazidime-avibactam be a way to avoid the need for combo therapy in the targeted treatment of deep-seated DTR Gram-negative infections?. Antimicrob Agents Chemother. 2023;67(11):e0096923. doi: 10.1128/aac.00969-23 37843260 PMC10648963

[38] Hepper HJ, Sieber C, Walger P, Bahrmann P, Singler K. Infections in the elderly. Crit Care Clin. 2013;29(3):757–74. doi: 10.1016/j.ccc.2013.03.016 23830661

[39] Nachamuthu S, Ibrahim NA, Su Min Hu F, Ganesh S. Carbapenem-resistant *Enterobacteriaceae Bacteraemia* in intensive care unit: A descriptive study on risk factors and clinical outcome. IMJM. 2023;22(4). doi: 10.31436/imjm.v22i4.2272

[40] Palacios-Baena ZR, Giannella M, Manissero D, Rodríguez-Baño J, Viale P, Lopes S, et al. Risk factors for carbapenem-resistant Gram-negative bacterial infections: A systematic review. Clin Microbiol Infect. 2021;27(2):228–35. doi: 10.1016/j.cmi.2020.10.016 33130270

[41] Van Der Wal HH, Van Deursen VM, Van Der Meer P, Voors AA. Comorbidities in heart failure. In: Bauersachs J, Butler J, Sandner P, editors. Heart failure. Cham: Springer International Publishing; 2017. 35–66. doi: 10.1007/164_2017_27 28382470

[42] Ferous S, Anastassopoulou C, Pitiriga V, Vrioni G, Tsakris A. Antimicrobial and diagnostic stewardship of the novel β-lactam/β-lactamase inhibitors for infections due to carbapenem-resistant Enterobacterales species and *Pseudomonas aeruginosa*. Antibiotics (Basel). 2024;13(3):285. doi: 10.3390/antibiotics13030285 38534720 PMC10967511

[43] Soriano A, Carmeli Y, Omrani AS, Moore LSP, Tawadrous M, Irani P. Ceftazidime-Avibactam for the treatment of serious gram-negative infections with limited treatment options: A systematic literature review. Infect Dis Ther. 2021;10(4):1989–2034. doi: 10.1007/s40121-021-00507-6 34379310 PMC8355581

