# Supplementary material for: Investigation on the use of ceftazidime-avibactam at a national hospital in Vietnam
Source: PLoS One. 2026 Oct 13;21(10):e0359631. doi: 10.1371/journal.pone.0359631 (PMC13660925; doi:10.1371/journal.pone.0359631)
Supplement: Supplementary file 2 [file pone.0359631.s004.docx]

**Response to Reviewers**

Dear Academic Editor and Reviewers,

We would like to thank the Academic Editor and Reviewers for their careful evaluation of our manuscript entitled “Investigation on the use of ceftazidime-avibactam at a national hospital in Vietnam”. We appreciate the constructive comments and suggestions, which have helped us improve the clarity, methodology, and interpretation of the manuscript.

We have revised the manuscript accordingly. Below, we provide a point-by-point response to each comment. Reviewer comments are presented in bold, followed by our responses and the corresponding revisions made in the manuscript.

Sincerely,

Quynh Thi Huong Bui,

On behalf of all authors

# **Response to Reviewer #1**

## **Comment 1. The Introduction should be expanded to include more detailed local epidemiological data regarding the types of infections analyzed in the study.**

**Response:** We agree that the previous Introduction did not describe the local epidemiology of the infection types included in our study. We have therefore expanded the Introduction by adding Vietnamese data on the distribution of major infection types, including respiratory tract infections, bloodstream infections and urinary tract infections.

**Revision made:** Page 5, Lines 66-72.

## **Comment 2. The clinical, microbiological characteristics and corresponding antimicrobial treatments of patients who did not achieve therapeutic success should be described more clearly, in greater detail, and in a more structured manner.**

**Response:** We have added a new table comparing patients with treatment success and those with treatment failure. This table includes relevant clinical characteristics, microbiological findings, and corresponding antimicrobial treatment regimens, allowing clearer characterization of patients who did not achieve therapeutic success. (Table 10)

**Revision made:** Page 19-20, Lines 220-227.

## **Comment 3. Lines 318–320: The authors are encouraged to further elaborate on the issue of dosing inadequacy in relation to patients’ renal function. Specifically, the administered dosing regimens should be explicitly reported and compared with those that would have been appropriate based on renal function in the individual cases.**

**Response:** Thank you for your suggestion. We have added a new table that compares the administered ceftazidime-avibactam regimen with the renal function-adjusted regimen that would have been appropriate for each dosing-inappropriate case. The table also reports the patients’ renal function category at ceftazidime-avibactam initiation. (Table 8)

**Revision made:** Page 18, Line 216.

## **Comment 4. Please indicate whether patients with augmented renal clearance were included, along with the dosing regimens administered in these cases. These data should also be contextualized in the Discussion, including an analysis of how many of these patients experienced therapeutic failure, in light of existing evidence in the literature (e.g., Tumbarello et al., Clin Infect Dis. 2021;73:1664–1676).**

**Response:** We reviewed renal function of patients and found one patient met this criterion, with an estimated CrCl of 208 mL/min. We have added this information to the Results section, including the ceftazidime-avibactam dosing regimen administered in this case and the corresponding treatment outcome. This patient experienced therapeutic failure, classified as worsened at discharge.

We have also revised the Discussion to contextualize this finding. Although no conclusion can be drawn from a single case, this may be clinically relevant for β-lactam therapy because increased renal clearance can reduce antibiotic exposure and potentially contribute to subtherapeutic concentrations.

**Revision made:** Page 20, Lines 228-230 and Page 29, Lines 393-396.

## **Comment 5. The Discussion would benefit from the inclusion of a dedicated section interpreting the study findings in the context of the most recent evidence on ceftazidime/avibactam use guided by Therapeutic Drug Monitoring.**

**Response:** We agree that the study findings should be interpreted in the context of emerging evidence on ceftazidime-avibactam optimization guided by therapeutic drug monitoring (TDM). However, in our hospital, ceftazidime-avibactam TDM was not available during the study period and we have acknowledged this as an important limitation of the study. Therefore, we have added a paragraph in the Discussion to describe how TDM may support ceftazidime-avibactam optimization.

**Revision made:** Page 28-29, Lines 380-388.

## **Comment 6. The resolution of the figures should be improved.**

**Response:** We have improved the resolution of all figures in the revised manuscript.

# **Response to Reviewer #2**

## **Comment 1. The definition of treatment success (cured/improved/alleviated) is subjective and requires clear objective criteria.**

**Response:** In our retrospective study, treatment outcome was extracted from the treating physicians’ documented assessment in the medical records at discharge. In routine hospital documentation, outcomes were recorded as cured, improved/alleviated, unchanged, worsened, or deceased. For analysis, cured and improved/alleviated were classified as treatment success, whereas unchanged, worsened, and deceased were classified as treatment failure.

We acknowledge that this outcome classification was based on physicians’ overall clinical assessment at discharge rather than predefined objective clinical, laboratory, or microbiological criteria. We have revised the Methods section to clarify how treatment outcomes were collected and classified.

**Revision made:** Page 7, Lines 109-114.

## **Comment 2. The multivariable logistic regression may be underpowered given the limited sample size and number of outcome events. Please justify model construction and assess potential overfitting.**

**Response:** We agree that the multivariable logistic regression may be underpowered because of the limited sample size and number of treatment-success events. To reduce the risk of overfitting, we used a multistep model-building approach, including univariate screening of candidate variables, multicollinearity assessment using VIF, and inclusion of eligible variables in the multivariable logistic regression model.

In addition, to reduce small-sample bias and assess the robustness of the findings, we performed Firth penalized logistic regression as a sensitivity analysis. According to this, the Methods, Results, and Discussion sections have been revised.

**Revision made:** Page 9, Lines 150-156; Page 20-23, Lines 231-253 and Page 30, Lines 405-414.

## **Comment 3. Important confounders such as ceftazidime-avibactam susceptibility, carbapenemase genotype, and appropriateness of therapy were not adequately explored in the regression analysis.**

**Response:** Ceftazidime-avibactam susceptibility and carbapenemase genotype data were not available for all 101 patients in our study. Therefore, these variables were not included in the regression analysis.

Appropriateness of ceftazidime-avibactam therapy was evaluated as a candidate predictor. However, it was not retained in the multivariable model because it did not meet the predefined criterion during univariate screening. We have revised the Results section to clarify the variable selection process.

**Revision made:** Page 21-23, Lines 233-253.

## **Comment 4. The microbiological methods are insufficiently described. Please specify susceptibility testing methods, breakpoints (CLSI/EUCAST), and carbapenemase detection techniques.**

**Response:** We have revised the Methods section to provide additional details on antimicrobial susceptibility testing, breakpoint interpretation, and carbapenemase detection.

**Revision made:** Page 7, Lines 100-107.

## **Comment 5. The conclusion that resistance developed rapidly after the introduction of ceftazidime-avibactam is not fully supported by this single-center cross-sectional study.**

**Response:** We agree that our single-center cross-sectional design does not allow us to conclude that ceftazidime-avibactam resistance has developed rapidly after its introduction in Vietnam. Our intention was to combine the relatively low ceftazidime-avibactam susceptibility observed in our study and findings of another Vietnamese study, such as the study by Trang PND, and then compare with international reports.

## **Comment 6. The study combines heterogeneous infection types (respiratory, bloodstream, urinary, intra-abdominal), which may influence treatment outcomes and limit interpretation of pooled analyses.**

**Response:** We agree that infection-specific analyses would provide a clearer interpretation of treatment outcomes. However, ceftazidime-avibactam is a newly introduced and high-cost antibiotic in Vietnam, resulting in a limited number of treated patients. Therefore, we did include all eligible infection types to describe real-world ceftazidime-avibactam use at our hospital. In the regression analysis, infection-type variables were considered as candidate predictors. However, these variables were not retained after multicollinearity assessment.

We respectfully ask the reviewer to accept this limitation of our study. As the use of ceftazidime-avibactam becomes more widespread in Vietnam and larger patient cohorts become available, we plan to conduct disease-specific analyses to better evaluate treatment effectiveness and outcomes for individual infection types.

## **Comment 7. The high proportion of combination therapy (>90%) is noteworthy, but its impact on treatment outcomes was not evaluated.**

**Response:** We agree that the high proportion of combination therapy is noteworthy. In our analysis, ceftazidime-avibactam monotherapy was evaluated as a candidate variable for treatment outcome. This indirectly assessed the potential impact of combination therapy, as patients not receiving ceftazidime-avibactam monotherapy were considered to have received ceftazidime-avibactam in combination with other antibiotics. However, according to our predefined regression process, candidate variables were first assessed using univariate logistic regression, and ceftazidime-avibactam monotherapy did not meet the criterion for inclusion in the multivariable model. Therefore, it was not retained for further regression analysis.

## **Comment 8. The rationale for carbapenemase genotyping in only 37 isolates should be clarified, including selection criteria and representativeness.**

**Response:** We agree that the rationale and selection criteria for carbapenemase genotyping should be clarified. In our hospital, carbapenemase genotyping was recommended according to the hospital protocol for isolates showing phenotypic resistance to at least one carbapenem on antimicrobial susceptibility testing. However, because this test was not reimbursed by health insurance, it was not routinely performed for all eligible isolates. Therefore, carbapenemase genotyping data were available for 37 isolates.

**Revision made:** Page 31, Lines 426-431.

## **Comment 9. The authors should consider discussing recent literature addressing the evolving role of ceftazidime-avibactam in carbapenem-resistant infections, including emerging resistance mechanisms and the potential need for adjunctive or combination therapies.**

**Response:** We have revised the Discussion and added a paragraph discussing key emerging resistance mechanisms, including MBL production and *bla*KPC mutations that may reduce avibactam activity. We also expanded the discussion on the potential need for adjunctive or combination treatment strategies in difficult-to-treat resistant infections, particularly for MBL-producing organisms, in line with current IDSA recommendations.

**Revision made:** Page 25-26, Lines 309-316.

## **Comment 10. Table 1 contains a likely error, as ICU and non-ICU percentages are both reported as 47.52%.**

**Response:** We have corrected the non-ICU percentage in Table 1 to 52.48%.

**Revision made:** Page 11, Lines 171.

## **Comment 11. References 14 and 20 appear to cite the same study and should be checked for duplication.**

**Response:** We have checked the reference list and confirmed that References 14 and 20 referred to the same study. The duplicate reference has been removed, and the in-text citations and reference numbering have been updated accordingly.
