# Supplementary material for: Investigation on the use of ceftazidime-avibactam at a national hospital in Vietnam
Source: PLoS One. 2026 Oct 13;21(10):e0359631. doi: 10.1371/journal.pone.0359631 (PMC13660925; doi:10.1371/journal.pone.0359631)
Supplement: Supplementary file 3 [file pone.0359631.s002.docx]

The manuscript addresses a highly topical and relevant subject, considering both the novelty of the pharmacological option investigated and the challenges associated with optimizing antimicrobial therapy in complex and difficult-to-treat infections such as those examined in the study by Khue Dang Minh Van et al. The data presented, although derived from a limited population, provide additional insights worthy of consideration. However, the limitations acknowledged by the authors substantially impact the robustness of the results and the conclusions drawn. Given the paucity of data available from the specific geographical area investigated, the manuscript may be considered suitable for publication, provided that several revisions and additions are addressed:

1.	The Introduction should be expanded to include more detailed local epidemiological data regarding the types of infections analyzed in the study.

2.	The clinical, microbiological characteristics and corresponding antimicrobial treatments of patients who did not achieve therapeutic success should be described more clearly, in greater detail, and in a more structured manner.

3.	Lines 318–320: The authors are encouraged to further elaborate on the issue of dosing inadequacy in relation to patients’ renal function. Specifically, the administered dosing regimens should be explicitly reported and compared with those that would have been appropriate based on renal function in the individual cases.

4.	Please indicate whether patients with augmented renal clearance were included, along with the dosing regimens administered in these cases. These data should also be contextualized in the Discussion, including an analysis of how many of these patients experienced therapeutic failure, in light of existing evidence in the literature (e.g., Tumbarello et al., Clin Infect Dis. 2021;73:1664–1676).

5.	The Discussion would benefit from the inclusion of a dedicated section interpreting the study findings in the context of the most recent evidence on ceftazidime/avibactam use guided by Therapeutic Drug Monitoring.

6.	The resolution of the figures should be improved.
