# Supplementary material for: Investigation on the use of ceftazidime-avibactam at a national hospital in Vietnam
Source: PLoS One. 2026 Oct 13;21(10):e0359631. doi: 10.1371/journal.pone.0359631 (PMC13660925; doi:10.1371/journal.pone.0359631)
Supplement: Supplementary file 4 [file pone.0359631.s005.docx]

**Response to Reviewers**

Dear Academic Editor and Reviewers,

We would like to thank the Academic Editor and Reviewers for their careful evaluation of our manuscript entitled “Investigation on the use of ceftazidime-avibactam at a national hospital in Vietnam”. We appreciate the constructive comments and suggestions, which have helped us improve the clarity, methodology, and interpretation of the manuscript.

We have revised the manuscript accordingly. Below, we provide a point-by-point response to each comment. Reviewer comments are presented in bold, followed by our responses and the corresponding revisions made in the manuscript.

Sincerely,

Quynh Thi Huong Bui,

On behalf of all authors

# **Response to Reviewer #2**

## **Comment 1. The Discussion should clearly distinguish that ICU admission remained statistically significant in both the multivariable and Firth penalized logistic regression analyses, whereas the association with heart failure became borderline in the sensitivity analysis. The conclusions should be slightly moderated to reflect this finding.**

**Response:** Thank you for this helpful comment.

We have revised the Discussion to further clarify this point:

- The association between comorbid heart failure and treatment success had already been described with caution, noting that it became borderline and was not confirmed as statistically significant in the Firth penalized logistic regression sensitivity analysis. (Page 30, Lines 408-417)
- We have now additionally clarified that ICU treatment remained statistically significant in both the multivariable logistic regression model and the Firth penalized logistic regression sensitivity analysis. (Page 29, Lines 397-402)

The Conclusion has also been revised to emphasize ICU treatment as the more robust finding and to clarify that the association with comorbid heart failure should be interpreted cautiously. (Page 31, Lines 444-449)

## **Comment 2. The Discussion would be strengthened by briefly emphasizing the implications of the findings for antimicrobial stewardship, particularly regarding timely microbiological diagnosis, appropriate renal dose adjustment, and judicious use of ceftazidime-avibactam to minimize the emergence of resistance.**

**Response:** Thank you for this helpful suggestion. We have revised the Discussion to briefly emphasize the antimicrobial stewardship implications of our findings. (Page 30, Lines 418-425)

## **Comment 3. The authors may consider citing the following recent publication while discussing antimicrobial stewardship and optimization of antimicrobial therapy, as it is relevant to the clinical implications of rational antimicrobial use: doi:10.4103/jfmpc.jfmpc_124_25.**

**Response:** Thank you for this suggestion. We carefully considered the recommended publication. However, after further evaluation, we decided not to include it as a reference in the revised manuscript, as its focus was not sufficiently aligned with the specific scope of our study. We appreciate the recommendation and may consider this article for our future studies that are more closely related to this topic.
