# Supplementary material for: Investigation on the use of ceftazidime-avibactam at a national hospital in Vietnam
Source: PLoS One. 2026 Oct 13;21(10):e0359631. doi: 10.1371/journal.pone.0359631 (PMC13660925; doi:10.1371/journal.pone.0359631)
Supplement: Peer review history

# Peer review: Decision Letter 0

**DOI:** 10.1371/journal.pone.0359631.r001

**Authors:** Sahjid Mukhida

21 Jun 2026

Dear Dr. Bui,

Thank you for submitting your manuscript to PLOS ONE. After careful consideration, we feel that it has merit but does not fully meet PLOS ONE’s publication criteria as it currently stands. Therefore, we invite you to submit a revised version of the manuscript that addresses the points raised during the review process.

Please submit your revised manuscript by Aug 05 2026 11:59PM. If you will need more time than this to complete your revisions, please reply to this message or contact the journal office at plosone@plos.org. When you're ready to submit your revision, log on to https://www.editorialmanager.com/pone/ and select the 'Submissions Needing Revision' folder to locate your manuscript file.

- A letter that responds to each point raised by the academic editor and reviewer(s). You should upload this letter as a separate file labeled 'Response to Reviewers'.
- A marked-up copy of your manuscript that highlights changes made to the original version. You should upload this as a separate file labeled 'Revised Manuscript with Track Changes'.
- An unmarked version of your revised paper without tracked changes. You should upload this as a separate file labeled 'Manuscript'.

If you would like to make changes to your financial disclosure, please include your updated statement in your cover letter. Guidelines for resubmitting your figure files are available below the reviewer comments at the end of this letter.

If applicable, we recommend that you deposit your laboratory protocols in protocols.io to enhance the reproducibility of your results. Protocols.io assigns your protocol its own identifier (DOI) so that it can be cited independently in the future. For instructions see: https://journals.plos.org/plosone/s/submission-guidelines#loc-laboratory-protocols. Additionally, PLOS ONE offers an option for publishing peer-reviewed Lab Protocol articles, which describe protocols hosted on protocols.io. Read more information on sharing protocols at https://plos.org/protocols?utm_medium=editorial-email&utm_source=authorletters&utm_campaign=protocols.

As the corresponding author, your ORCID iD is verified in the submission system and will appear in the published article. PLOS supports the use of ORCID, and we encourage all coauthors to register for an ORCID iD and use it as well. Please encourage your coauthors to verify their ORCID iD within the submission system before final acceptance, as unverified ORCID iDs will not appear in the published article. *Only*  the individual author can complete the verification step; PLOS staff *cannot*  verify ORCID iDs on behalf of authors.

We look forward to receiving your revised manuscript.

Kind regards,

Sahjid Mukhida

Academic Editor

PLOS One

Journal requirements:

When submitting your revision, we need you to address these additional requirements.

1. Please ensure that your manuscript meets PLOS ONE's style requirements, including those for file naming. The PLOS ONE style templates can be found at

https://journals.plos.org/plosone/s/file?id=wjVg/PLOSOne_formatting_sample_main_body.pdf and

https://journals.plos.org/plosone/s/file?id=ba62/PLOSOne_formatting_sample_title_authors_affiliations.pdf

2. Please include a separate caption for each figure in your manuscript.

If the reviewer comments include a recommendation to cite specific previously published works, please review and evaluate these publications to determine whether they are relevant and should be cited. There is no requirement to cite these works unless the editor has indicated otherwise.

Additional Editor Comments:

Dear Authors,

Both reviewers recognize the importance and relevance of your study, particularly in the context of antimicrobial stewardship and the limited available data from the study region. Reviewer 1 considers the manuscript potentially suitable for publication pending revision, while Reviewer 2 highlights several important methodological and interpretative concerns.

After careful evaluation, the manuscript requires major revision before it can be considered further.

The authors are requested to carefully address all reviewers’ comments in a point-by-point manner and revise the manuscript accordingly. In particular, attention should be given to:

Clarification and justification of outcome definitions

Detailed description and, where necessary, revision of microbiological methods

Review and possible reanalysis of regression modeling and statistical approaches

More cautious and accurate interpretation of antimicrobial resistance findings

Strengthening the discussion of limitations and ensuring conclusions are appropriately tempered

Please ensure that all changes are clearly marked in the revised manuscript and that a detailed response letter is submitted addressing each comment raised by the reviewers.

We look forward to receiving your revised submission.

Sincerely,

[Note: HTML markup is below. Please do not edit.]

Reviewers' comments:

Reviewer's Responses to Questions

**Comments to the Author**

1. Is the manuscript technically sound, and do the data support the conclusions?

Reviewer #1: Partly

Reviewer #2: Yes

**********

2. Has the statistical analysis been performed appropriately and rigorously?

Reviewer #1: I Don't Know

Reviewer #2: Yes

**********

3. Have the authors made all data underlying the findings in their manuscript fully available?

The PLOS Data policy

Reviewer #1: Yes

Reviewer #2: Yes

**********

4. Is the manuscript presented in an intelligible fashion and written in standard English?

Reviewer #1: Yes

Reviewer #2: Yes

**********

Reviewer #1: The manuscript addresses a highly topical and relevant subject, considering both the novelty of the pharmacological option investigated and the challenges associated with optimizing antimicrobial therapy in complex and difficult-to-treat infections such as those examined in the study by Khue Dang Minh Van et al. The data presented, although derived from a limited population, provide additional insights worthy of consideration. However, the limitations acknowledged by the authors substantially impact the robustness of the results and the conclusions drawn. Given the paucity of data available from the specific geographical area investigated, the manuscript may be considered suitable for publication, provided that several revisions and additions are addressed (see file attached)

Reviewer #2: This study addresses an important antimicrobial stewardship topic and provides local data on ceftazidime-avibactam use in Vietnam. However, there are several methodological concerns, particularly regarding outcome definition, microbiological methods, regression modeling, and interpretation of resistance data. I recommend Major Revision before the manuscript can be considered for publication.

1. The definition of treatment success (cured/improved/alleviated) is subjective and requires clear objective criteria.

2. The multivariable logistic regression may be underpowered given the limited sample size and number of outcome events. Please justify model construction and assess potential overfitting.

3. Important confounders such as ceftazidime-avibactam susceptibility, carbapenemase genotype, and appropriateness of therapy were not adequately explored in the regression analysis.

4. The microbiological methods are insufficiently described. Please specify susceptibility testing methods, breakpoints (CLSI/EUCAST), and carbapenemase detection techniques.

5. The conclusion that resistance developed rapidly after the introduction of ceftazidime-avibactam is not fully supported by this single-center cross-sectional study.

6. The study combines heterogeneous infection types (respiratory, bloodstream, urinary, intra-abdominal), which may influence treatment outcomes and limit interpretation of pooled analyses.

7. The high proportion of combination therapy (>90%) is noteworthy, but its impact on treatment outcomes was not evaluated.

8. The rationale for carbapenemase genotyping in only 37 isolates should be clarified, including selection criteria and representativeness.

9. The authors should consider discussing recent literature addressing the evolving role of ceftazidime-avibactam in carbapenem-resistant infections, including emerging resistance mechanisms and the potential need for adjunctive or combination therapies.

10. Table 1 contains a likely error, as ICU and non-ICU percentages are both reported as 47.53%.

11. References 14 and 20 appear to cite the same study and should be checked for duplication.

**********

what does this mean?). If published, this will include your full peer review and any attached files.

If you choose “no”, your identity will remain anonymous but your review may still be made public.

**Do you want your identity to be public for this peer review?**  For information about this choice, including consent withdrawal, please see our Privacy Policy

Reviewer #1: No

Reviewer #2: **Yes:**  Dr. Nikunja Kumar Das

**********

[NOTE: If reviewer comments were submitted as an attachment file, they will be attached to this email and accessible via the submission site. Please log into your account, locate the manuscript record, and check for the action link "View Attachments". If this link does not appear, there are no attachment files.]

To ensure your figures meet our technical requirements, please review our figure guidelines: https://journals.plos.org/plosone/s/figures

You may also use PLOS’s free figure tool, NAAS, to help you prepare publication quality figures: https://journals.plos.org/plosone/s/figures#loc-tools-for-figure-preparation.

NAAS will assess whether your figures meet our technical requirements by comparing each figure against our figure specifications.

Attachment Submitted filename: ReviewerJAPLOSONE.docx

# Author comment: Author response to Decision Letter 1

**DOI:** 10.1371/journal.pone.0359631.r002

3 Aug 2026

Response to Reviewers

Dear Academic Editor and Reviewers,

We would like to thank the Academic Editor and Reviewers for their careful evaluation of our manuscript entitled “Investigation on the use of ceftazidime-avibactam at a national hospital in Vietnam”. We appreciate the constructive comments and suggestions, which have helped us improve the clarity, methodology, and interpretation of the manuscript.

We have revised the manuscript accordingly. Below, we provide a point-by-point response to each comment. Reviewer comments are presented in bold, followed by our responses and the corresponding revisions made in the manuscript.

Sincerely,

Quynh Thi Huong Bui,

On behalf of all authors

Response to Reviewer #1

Comment 1. The Introduction should be expanded to include more detailed local epidemiological data regarding the types of infections analyzed in the study.

Response: We agree that the previous Introduction did not describe the local epidemiology of the infection types included in our study. We have therefore expanded the Introduction by adding Vietnamese data on the distribution of major infection types, including respiratory tract infections, bloodstream infections and urinary tract infections.

Revision made: Page 5, Lines 66-72.

Comment 2. The clinical, microbiological characteristics and corresponding antimicrobial treatments of patients who did not achieve therapeutic success should be described more clearly, in greater detail, and in a more structured manner.

Response: We have added a new table comparing patients with treatment success and those with treatment failure. This table includes relevant clinical characteristics, microbiological findings, and corresponding antimicrobial treatment regimens, allowing clearer characterization of patients who did not achieve therapeutic success. (Table 10)

Revision made: Page 19-20, Lines 219-226.

Comment 3. Lines 318–320: The authors are encouraged to further elaborate on the issue of dosing inadequacy in relation to patients’ renal function. Specifically, the administered dosing regimens should be explicitly reported and compared with those that would have been appropriate based on renal function in the individual cases.

Response: Thank you for your suggestion. We have added a new table that compares the administered ceftazidime-avibactam regimen with the renal function-adjusted regimen that would have been appropriate for each dosing-inappropriate case. The table also reports the patients’ renal function category at ceftazidime-avibactam initiation. (Table 8)

Revision made: Page 18, Line 215.

Comment 4. Please indicate whether patients with augmented renal clearance were included, along with the dosing regimens administered in these cases. These data should also be contextualized in the Discussion, including an analysis of how many of these patients experienced therapeutic failure, in light of existing evidence in the literature (e.g., Tumbarello et al., Clin Infect Dis. 2021;73:1664–1676).

Response: We reviewed renal function of patients and found one patient met this criterion, with an estimated CrCl of 208 mL/min. We have added this information to the Results section, including the ceftazidime-avibactam dosing regimen administered in this case and the corresponding treatment outcome. This patient experienced therapeutic failure, classified as worsened at discharge.

We have also revised the Discussion to contextualize this finding. Although no conclusion can be drawn from a single case, this may be clinically relevant for β-lactam therapy because increased renal clearance can reduce antibiotic exposure and potentially contribute to subtherapeutic concentrations.

Revision made: Page 20, Lines 227-229 and Page 29, Lines 392-395.

Comment 5. The Discussion would benefit from the inclusion of a dedicated section interpreting the study findings in the context of the most recent evidence on ceftazidime/avibactam use guided by Therapeutic Drug Monitoring.

Response: We agree that the study findings should be interpreted in the context of emerging evidence on ceftazidime-avibactam optimization guided by therapeutic drug monitoring (TDM). However, in our hospital, ceftazidime-avibactam TDM was not available during the study period and we have acknowledged this as an important limitation of the study. Therefore, we have added a paragraph in the Discussion to describe how TDM may support ceftazidime-avibactam optimization.

Revision made: Page 28-29, Lines 379-387.

Comment 6. The resolution of the figures should be improved.

Response: We have improved the resolution of all figures in the revised manuscript.

Response to Reviewer #2

Comment 1. The definition of treatment success (cured/improved/alleviated) is subjective and requires clear objective criteria.

Response: In our retrospective study, treatment outcome was extracted from the treating physicians’ documented assessment in the medical records at discharge. In routine hospital documentation, outcomes were recorded as cured, improved/alleviated, unchanged, worsened, or deceased. For analysis, cured and improved/alleviated were classified as treatment success, whereas unchanged, worsened, and deceased were classified as treatment failure.

We acknowledge that this outcome classification was based on physicians’ overall clinical assessment at discharge rather than predefined objective clinical, laboratory, or microbiological criteria. We have revised the Methods section to clarify how treatment outcomes were collected and classified.

Revision made: Page 7, Lines 109-114.

Comment 2. The multivariable logistic regression may be underpowered given the limited sample size and number of outcome events. Please justify model construction and assess potential overfitting.

Response: We agree that the multivariable logistic regression may be underpowered because of the limited sample size and number of treatment-success events. To reduce the risk of overfitting, we used a multistep model-building approach, including univariate screening of candidate variables, multicollinearity assessment using VIF, and inclusion of eligible variables in the multivariable logistic regression model.

In addition, to reduce small-sample bias and assess the robustness of the findings, we performed Firth penalized logistic regression as a sensitivity analysis. According to this, the Methods, Results, and Discussion sections have been revised.

Revision made: Page 9, Lines 150-156; Page 20-23, Lines 230-252 and Page 30, Lines 404-413.

Comment 3. Important confounders such as ceftazidime-avibactam susceptibility, carbapenemase genotype, and appropriateness of therapy were not adequately explored in the regression analysis.

Response: Ceftazidime-avibactam susceptibility and carbapenemase genotype data were not available for all 101 patients in our study. Therefore, these variables were not included in the regression analysis.

Appropriateness of ceftazidime-avibactam therapy was evaluated as a candidate predictor. However, it was not retained in the multivariable model because it did not meet the predefined criterion during univariate screening. We have revised the Results section to clarify the variable selection process.

Revision made: Page 21-23, Lines 232-252.

Comment 4. The microbiological methods are insufficiently described. Please specify susceptibility testing methods, breakpoints (CLSI/EUCAST), and carbapenemase detection techniques.

Response: We have revised the Methods section to provide additional details on antimicrobial susceptibility testing, breakpoint interpretation, and carbapenemase detection.

Revision made: Page 7, Lines 100-107.

Comment 5. The conclusion that resistance developed rapidly after the introduction of ceftazidime-avibactam is not fully supported by this single-center cross-sectional study.

Response: We agree that our single-center cross-sectional design does not allow us to conclude that ceftazidime-avibactam resistance has developed rapidly after its introduction in Vietnam. Our intention was to combine the relatively low ceftazidime-avibactam susceptibility observed in our study and findings of another Vietnamese study, such as the study by Trang PND, and then compare with international reports.

Comment 6. The study combines heterogeneous infection types (respiratory, bloodstream, urinary, intra-abdominal), which may influence treatment outcomes and limit interpretation of pooled analyses.

Response: We agree that infection-specific analyses would provide a clearer interpretation of treatment outcomes. However, ceftazidime-avibactam is a newly introduced and high-cost antibiotic in Vietnam, resulting in a limited number of treated patients. Therefore, we did include all eligible infection types to describe real-world ceftazidime-avibactam use at our hospital. In the regression analysis, infection-type variables were considered as candidate predictors. However, these variables were not retained after multicollinearity assessment.

We respectfully ask the reviewer to accept this limitation of our study. As the use of ceftazidime-avibactam becomes more widespread in Vietnam and larger patient cohorts become available, we plan to conduct disease-specific analyses to better evaluate treatment effectiveness and outcomes for individual infection types.

Comment 7. The high proportion of combination therapy (>90%) is noteworthy, but its impact on treatment outcomes was not evaluated.

Response: We agree that the high proportion of combination therapy is noteworthy. In our analysis, ceftazidime-avibactam monotherapy was evaluated as a candidate variable for treatment outcome. This indirectly assessed the potential impact of combination therapy, as patients not receiving ceftazidime-avibactam monotherapy were considered to have received ceftazidime-avibactam in combination with other antibiotics. However, according to our predefined regression process, candidate variables were first assessed using univariate logistic regression, and ceftazidime-avibactam monotherapy did not meet the criterion for inclusion in the multivariable model. Therefore, it was not retained for further regression analysis.

Comment 8. The rationale for carbapenemase genotyping in only 37 isolates should be clarified, including selection criteria and representativeness.

Response: We agree that the rationale and selection criteria for carbapenemase genotyping should be clarified. In our hospital, carbapenemase genotyping was recommended according to the hospital protocol for isolates showing phenotypic resistance to at least one carbapenem on antimicrobial susceptibility testing. However, because this test was not reimbursed by health insurance, it was not routinely performed for all eligible isolates. Therefore, carbapenemase genotyping data were available for 37 isolates.

Revision made: Page 31, Lines 425-430.

Comment 9. The authors should consider discussing recent literature addressing the evolving role of ceftazidime-avibactam in carbapenem-resistant infections, including emerging resistance mechanisms and the potential need for adjunctive or combination therapies.

Response: We have revised the Discussion and added a paragraph discussing key emerging resistance mechanisms, including MBL production and blaKPC mutations that may reduce avibactam activity. We also expanded the discussion on the potential need for adjunctive or combination treatment strategies in difficult-to-treat resistant infections, particularly for MBL-producing organisms, in line with current IDSA recommendations.

Revision made: Page 25-26, Lines 308-315.

Comment 10. Table 1 contains a likely error, as ICU and non-ICU percentages are both reported as 47.52%.

Response: We have corrected the non-ICU percentage in Table 1 to 52.48%.

Revision made: Page 11, Lines 171.

Comment 11. References 14 and 20 appear to cite the same study and should be checked for duplication.

Response: We have checked the reference list and confirmed that References 14 and 20 referred to the same study. The duplicate reference has been removed, and the in-text citations and reference numbering have been updated accordingly.

Attachment Submitted filename: Response to Reviewers.docx

# Peer review: Decision Letter 1

**DOI:** 10.1371/journal.pone.0359631.r003

**Authors:** Sahjid Mukhida

9 Sep 2026

Dear Dr. Bui,

Thank you for submitting your manuscript to PLOS One. After careful consideration, we feel that it has merit but does not fully meet PLOS One’s publication criteria as it currently stands. Therefore, we invite you to submit a revised version of the manuscript that addresses the points raised during the review process.

Please submit your revised manuscript by Oct 24 2026 11:59PM. If you will need more time than this to complete your revisions, please reply to this message or contact the journal office at plosone@plos.org. When you're ready to submit your revision, log on to https://www.editorialmanager.com/pone/ and select the 'Submissions Needing Revision' folder to locate your manuscript file.

- A letter that responds to each point raised by the academic editor and reviewer(s). You should upload this letter as a separate file labeled 'Response to Reviewers'.
- A marked-up copy of your manuscript that highlights changes made to the original version. You should upload this as a separate file labeled 'Revised Manuscript with Track Changes'.
- An unmarked version of your revised paper without tracked changes. You should upload this as a separate file labeled 'Manuscript'.

If you would like to make changes to your financial disclosure, please include your updated statement in your cover letter. Guidelines for resubmitting your figure files are available below the reviewer comments at the end of this letter.

If applicable, we recommend that you deposit your laboratory protocols in protocols.io to enhance the reproducibility of your results. Protocols.io assigns your protocol its own identifier (DOI) so that it can be cited independently in the future. For instructions see: https://journals.plos.org/plosone/s/submission-guidelines#loc-laboratory-protocols. Additionally, PLOS One offers an option for publishing peer-reviewed Lab Protocol articles, which describe protocols hosted on protocols.io. Read more information on sharing protocols at https://plos.org/protocols?utm_medium=editorial-email&utm_source=authorletters&utm_campaign=protocols.

As the corresponding author, your ORCID iD is verified in the submission system and will appear in the published article. PLOS supports the use of ORCID, and we encourage all coauthors to register for an ORCID iD and use it as well. Please encourage your coauthors to verify their ORCID iD within the submission system before final acceptance, as unverified ORCID iDs will not appear in the published article. *Only*  the individual author can complete the verification step; PLOS staff *cannot*  verify ORCID iDs on behalf of authors.

We look forward to receiving your revised manuscript.

Kind regards,

Sahjid Mukhida

Academic Editor

PLOS One

Journal Requirements:

If the reviewer comments include a recommendation to cite specific previously published works, please review and evaluate these publications to determine whether they are relevant and should be cited. There is no requirement to cite these works unless the editor has indicated otherwise.

Please review your reference list to ensure that it is complete and correct. If you have cited papers that have been retracted, please include the rationale for doing so in the manuscript text, or remove these references and replace them with relevant current references. Any changes to the reference list should be mentioned in the rebuttal letter that accompanies your revised manuscript. If you need to cite a retracted article, indicate the article’s retracted status in the References list and also include a citation and full reference for the retraction notice.

[Note: HTML markup is below. Please do not edit.]

Reviewers' comments:

Reviewer's Responses to Questions

**Comments to the Author**

Reviewer #2: All comments have been addressed

Reviewer #3: All comments have been addressed

**********

2. Is the manuscript technically sound, and do the data support the conclusions?

Reviewer #2: Yes

Reviewer #3: Yes

**********

3. Has the statistical analysis been performed appropriately and rigorously?

Reviewer #2: No

Reviewer #3: Yes

**********

4. Have the authors made all data underlying the findings in their manuscript fully available?

The PLOS Data policy

Reviewer #2: Yes

Reviewer #3: Yes

**********

5. Is the manuscript presented in an intelligible fashion and written in standard English?

Reviewer #2: Yes

Reviewer #3: Yes

**********

Reviewer #2: The authors have satisfactorily addressed the major concerns raised in the previous review, and the manuscript has improved considerably in terms of methodology, statistical analysis, and discussion. The study is clinically relevant and suitable for publication after addressing the following minor comments.

The Discussion should clearly distinguish that ICU admission remained statistically significant in both the multivariable and Firth penalized logistic regression analyses, whereas the association with heart failure became borderline in the sensitivity analysis. The conclusions should be slightly moderated to reflect this finding.

The Discussion would be strengthened by briefly emphasizing the implications of the findings for antimicrobial stewardship, particularly regarding timely microbiological diagnosis, appropriate renal dose adjustment, and judicious use of ceftazidime-avibactam to minimize the emergence of resistance.

The authors may consider citing the following recent publication while discussing antimicrobial stewardship and optimization of antimicrobial therapy, as it is relevant to the clinical implications of rational antimicrobial use: doi:10.4103/jfmpc.jfmpc_124_25.

Overall, I believe the manuscript is suitable for publication after these minor revisions.

Reviewer #3: The authors have addressed the issues and comments posed by the reviewer adequately and clarified the queries posed. They have added tables to include necessary variables. They have made the necessary modifications in the revised manuscript.

**********

what does this mean?). If published, this will include your full peer review and any attached files.

If you choose “no”, your identity will remain anonymous but your review may still be made public.

**Do you want your identity to be public for this peer review?**  For information about this choice, including consent withdrawal, please see our Privacy Policy

Reviewer #2: **Yes:**  Dr Nikunja Kumar Das

Reviewer #3: No

**********

[NOTE: If reviewer comments were submitted as an attachment file, they will be attached to this email and accessible via the submission site. Please log into your account, locate the manuscript record, and check for the action link "View Attachments". If this link does not appear, there are no attachment files.]

To ensure your figures meet our technical requirements, please review our figure guidelines: https://journals.plos.org/plosone/s/figures

You may also use PLOS’s free figure tool, NAAS, to help you prepare publication quality figures: https://journals.plos.org/plosone/s/figures#loc-tools-for-figure-preparation.

NAAS will assess whether your figures meet our technical requirements by comparing each figure against our figure specifications.

# Author comment: Author response to Decision Letter 2

**DOI:** 10.1371/journal.pone.0359631.r004

15 Sep 2026

Response to Reviewers

Dear Academic Editor and Reviewers,

We would like to thank the Academic Editor and Reviewers for their careful evaluation of our manuscript entitled “Investigation on the use of ceftazidime-avibactam at a national hospital in Vietnam”. We appreciate the constructive comments and suggestions, which have helped us improve the clarity, methodology, and interpretation of the manuscript.

We have revised the manuscript accordingly. Below, we provide a point-by-point response to each comment. Reviewer comments are presented in bold, followed by our responses and the corresponding revisions made in the manuscript.

Sincerely,

Quynh Thi Huong Bui,

On behalf of all authors

Response to Reviewer #2

Comment 1. The Discussion should clearly distinguish that ICU admission remained statistically significant in both the multivariable and Firth penalized logistic regression analyses, whereas the association with heart failure became borderline in the sensitivity analysis. The conclusions should be slightly moderated to reflect this finding.

Response: Thank you for this helpful comment.

We have revised the Discussion to further clarify this point:

• The association between comorbid heart failure and treatment success had already been described with caution, noting that it became borderline and was not confirmed as statistically significant in the Firth penalized logistic regression sensitivity analysis. (Page 30, Lines 408-417)

• We have now additionally clarified that ICU treatment remained statistically significant in both the multivariable logistic regression model and the Firth penalized logistic regression sensitivity analysis. (Page 29, Lines 397-402)

The Conclusion has also been revised to emphasize ICU treatment as the more robust finding and to clarify that the association with comorbid heart failure should be interpreted cautiously. (Page 31, Lines 444-449)

Comment 2. The Discussion would be strengthened by briefly emphasizing the implications of the findings for antimicrobial stewardship, particularly regarding timely microbiological diagnosis, appropriate renal dose adjustment, and judicious use of ceftazidime-avibactam to minimize the emergence of resistance.

Response: Thank you for this helpful suggestion. We have revised the Discussion to briefly emphasize the antimicrobial stewardship implications of our findings. (Page 30, Lines 418-425)

Comment 3. The authors may consider citing the following recent publication while discussing antimicrobial stewardship and optimization of antimicrobial therapy, as it is relevant to the clinical implications of rational antimicrobial use: doi:10.4103/jfmpc.jfmpc_124_25.

Response: Thank you for this suggestion. We carefully considered the recommended publication. However, after further evaluation, we decided not to include it as a reference in the revised manuscript, as its focus was not sufficiently aligned with the specific scope of our study. We appreciate the recommendation and may consider this article for our future studies that are more closely related to this topic.

Attachment Submitted filename: Response_to_Reviewers_auresp_2.docx

# Editor report: Decision Letter 2

**DOI:** 10.1371/journal.pone.0359631.r005

**Authors:** Sahjid Mukhida

16 Sep 2026

Investigation on the use of ceftazidime-avibactam at a national hospital in Vietnam

PONE-D-26-10894R2

Dear Dr. Bui,

We’re pleased to inform you that your manuscript has been judged scientifically suitable for publication and will be formally accepted for publication once it meets all outstanding technical requirements.

Within one week, you’ll receive an e-mail detailing the required amendments. When these have been addressed, you’ll receive a formal acceptance letter and your manuscript will be scheduled for publication.

An invoice will be generated when your article is formally accepted. Please note, if your institution has a publishing partnership with PLOS and your article meets the relevant criteria, all or part of your publication costs will be covered. Please make sure your user information is up-to-date by logging into Editorial Manager at Editorial Manager® and clicking the ‘Update My Information' link at the top of the page. For questions related to billing, please contact billing support.

If your institution or institutions have a press office, please notify them about your upcoming paper to help maximize its impact. If they’ll be preparing press materials, please inform our press team as soon as possible -- no later than 48 hours after receiving the formal acceptance. Your manuscript will remain under strict press embargo until 2 pm Eastern Time on the date of publication. For more information, please contact onepress@plos.org.

Kind regards,

Sahjid Mukhida

Academic Editor

PLOS One

Additional Editor Comments (optional):

I have gone through the revised manuscript and found satisfactory with reviewer's comment.

Reviewers' comments:

As there were minor revision, i have checked and found satisfactory with all reviewer's comment

# Editor report: Acceptance letter

**DOI:** 10.1371/journal.pone.0359631.r006

**Authors:** Sahjid Mukhida

PONE-D-26-10894R2

PLOS One

Dear Dr. Bui,

I'm pleased to inform you that your manuscript has been deemed suitable for publication in PLOS One. Congratulations! Your manuscript is now being handed over to our production team.

At this stage, our production department will prepare your paper for publication. This includes ensuring the following:

* All references, tables, and figures are properly cited

* All relevant supporting information is included in the manuscript submission,

* There are no issues that prevent the paper from being properly typeset

You will receive further instructions from the production team, including instructions on how to review your proof when it is ready. Please keep in mind that we are working through a large volume of accepted articles, so please give us a few days to review your paper and let you know the next and final steps.

Lastly, if your institution or institutions have a press office, please let them know about your upcoming paper now to help maximize its impact. If they'll be preparing press materials, please inform our press team within the next 48 hours. Your manuscript will remain under strict press embargo until 2 pm Eastern Time on the date of publication. For more information, please contact onepress@plos.org.

You will receive an invoice from PLOS for your publication fee after your manuscript has reached the completed accept phase. If you receive an email requesting payment before acceptance or for any other service, this may be a phishing scheme. Learn how to identify phishing emails and protect your accounts at https://explore.plos.org/phishing.

If we can help with anything else, please email us at customercare@plos.org.

Thank you for submitting your work to PLOS One and supporting open access.

Kind regards,

PLOS One Editorial Office Staff

on behalf of

Dr. Sahjid Mukhida

Academic Editor

PLOS One
